# Supplementary material for: DISSECTING TUMOR TRANSCRIPTIONAL HETEROGENEITY FROM SINGLE-CELL RNA-SEQ DATA BY GENERALIZED BINARY COVARIANCE DECOMPOSITION
Source: bioRxiv. 2023 Aug 17:2023.08.15.553436. Preprint. [Version 1] doi: 10.1101/2023.08.15.553436 (PMC10462040; doi:10.1101/2023.08.15.553436)
Supplement: Supplement 1 [file media-1.pdf]

Supplementary Figures for "Dissecting tumor transcriptional heterogeneity from single-cell RNA-seq data by generalized binary covariance decomposition"

Yusha Liu[1], Peter Carbonetto[1,2], Jason Willwerscheid[3], Scott A. Oakes[4], Kay F. Macleod[5], and Matthew Stephens[1,6]

[1]Department of Human Genetics, The University of Chicago, Chicago, IL, USA
[2]Research Computing Center, The University of Chicago, Chicago, IL, USA
[3]Department of Mathematics and Computer Science, Providence College, Providence, RI, USA
[4]Department of Pathology, University of Chicago, Chicago, IL, USA
[5]Ben May Department for Cancer Research, University of Chicago, Chicago, IL, USA
[6]Department of Statistics, The University of Chicago, Chicago, IL, USA

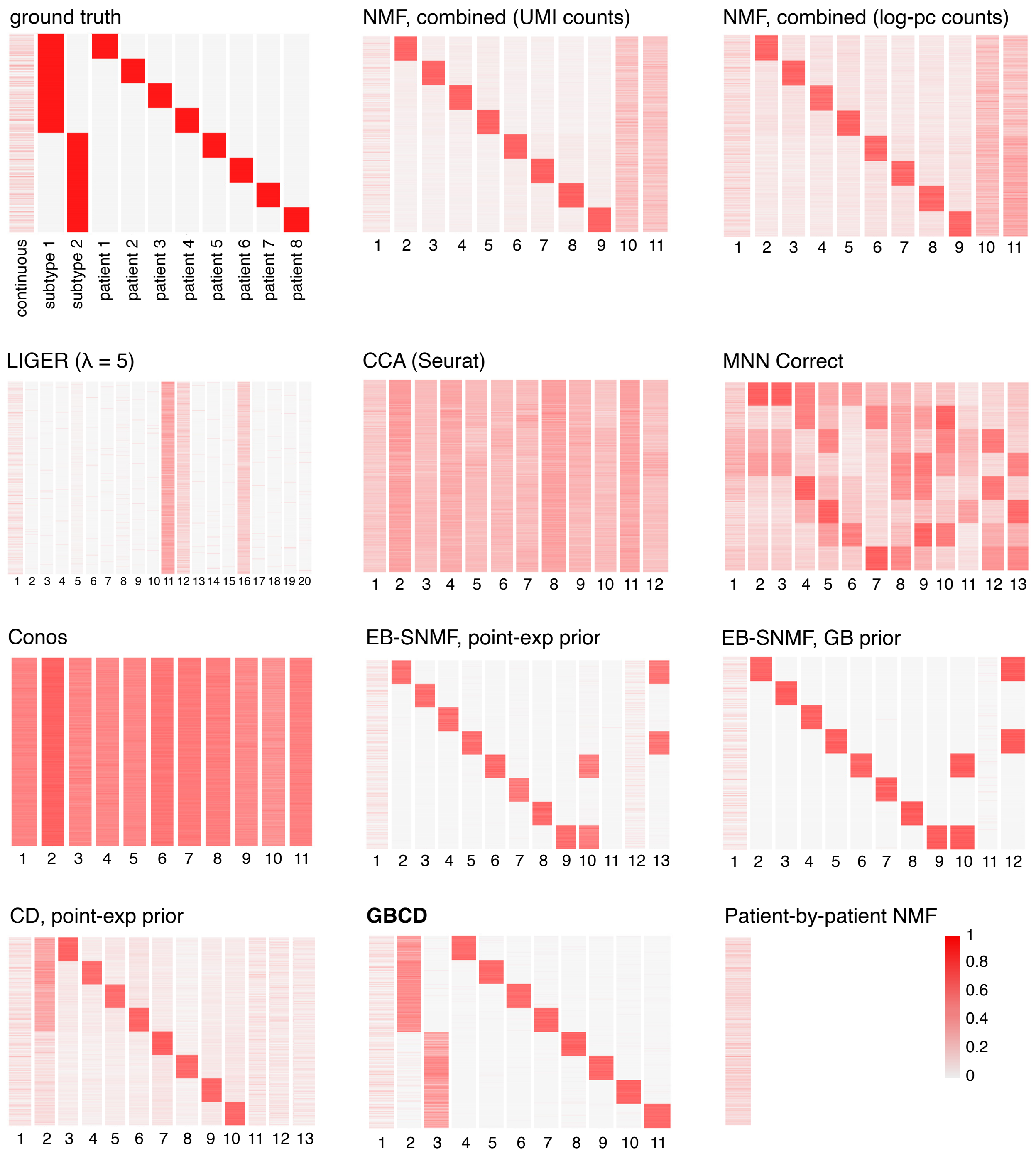

Figure S1: GEP membership estimates for one of the 20 simulated data sets. In each heatmap, rows are cells, columns are GEPs. The columns were scaled separately so that the largest value in each column was always 1. Note that all methods identified one or more components that were strongly correlated with cellular detection rate; these components were not included in the heatmaps. Also note the patient-by-patient NMF identified only a single GEP.

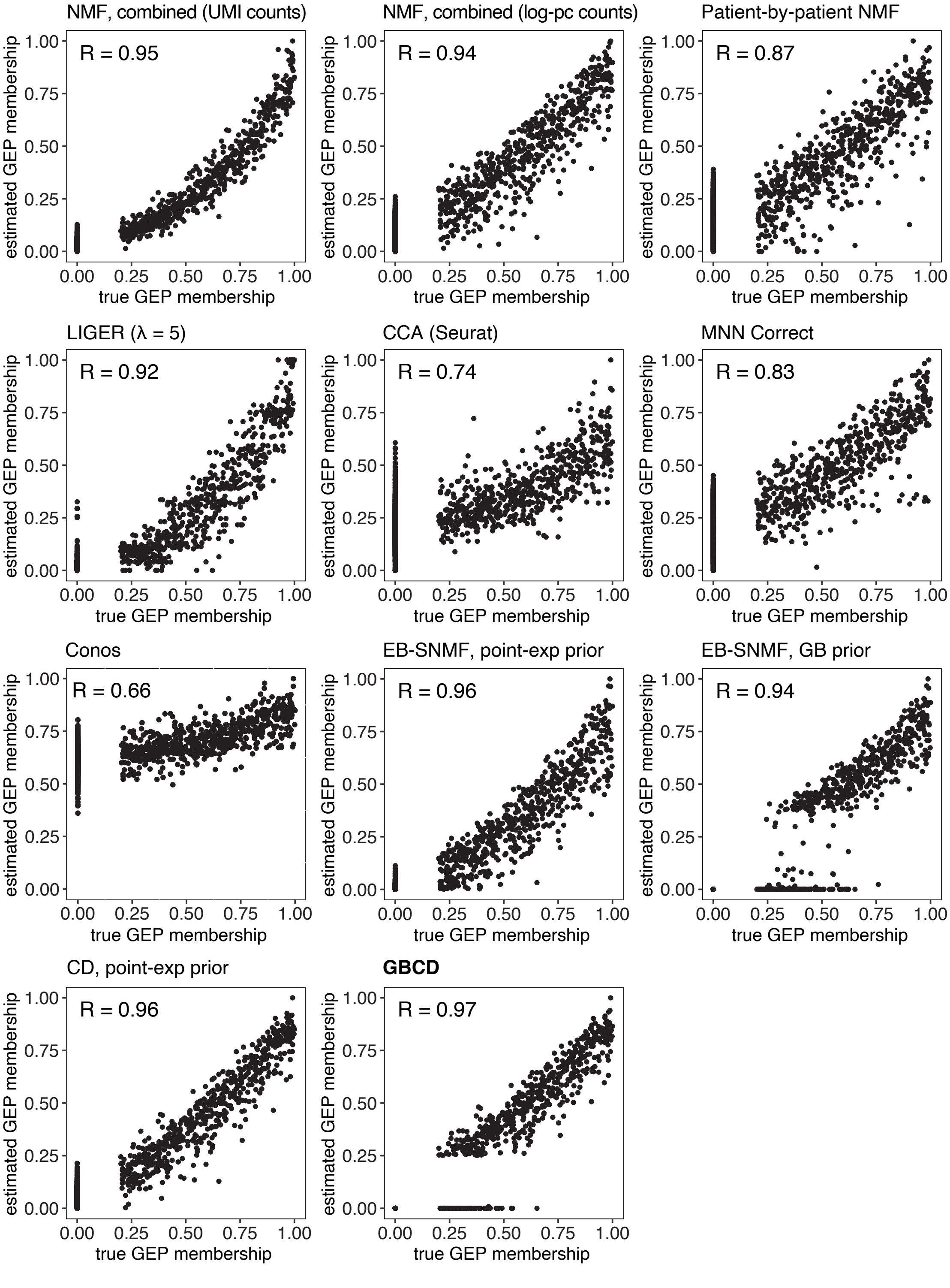

Figure S2: GEP membership estimates from Supplementary Fig. S1 are shown in more detail for the continuous GEP only. The scatterplots compare the true and estimated memberships for each method. For each plot, the membership values are rescaled so that the maximum membership is always 1.

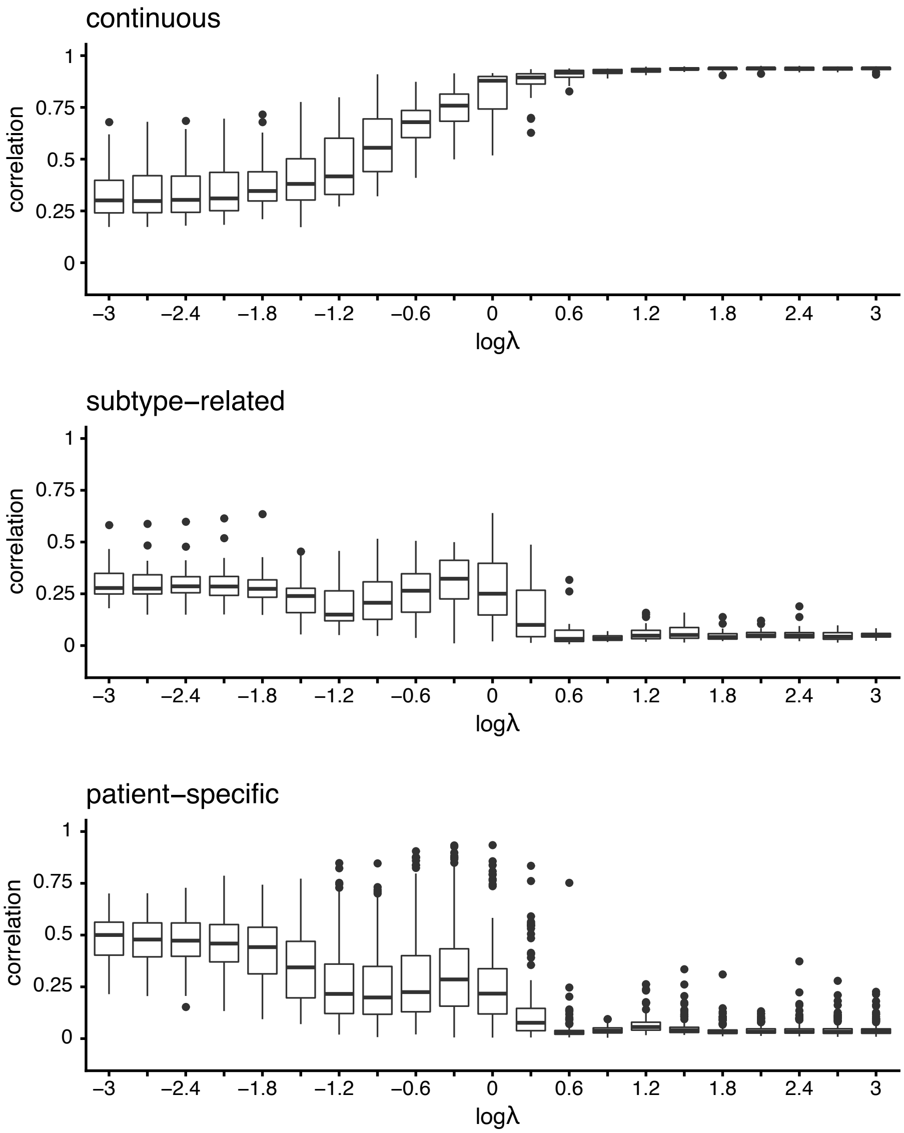

Figure S3: Performance of LIGER in recovering the continuous, subtype-related and patient-specific GEPs across all 20 simulated data sets as the regularization parameter λ was varied. For each true GEP, accuracy of the LIGER's estimate was measured using the highest Pearson correlation between the true GEP membership and the estimated membership among all GEPs identified by LIGER.

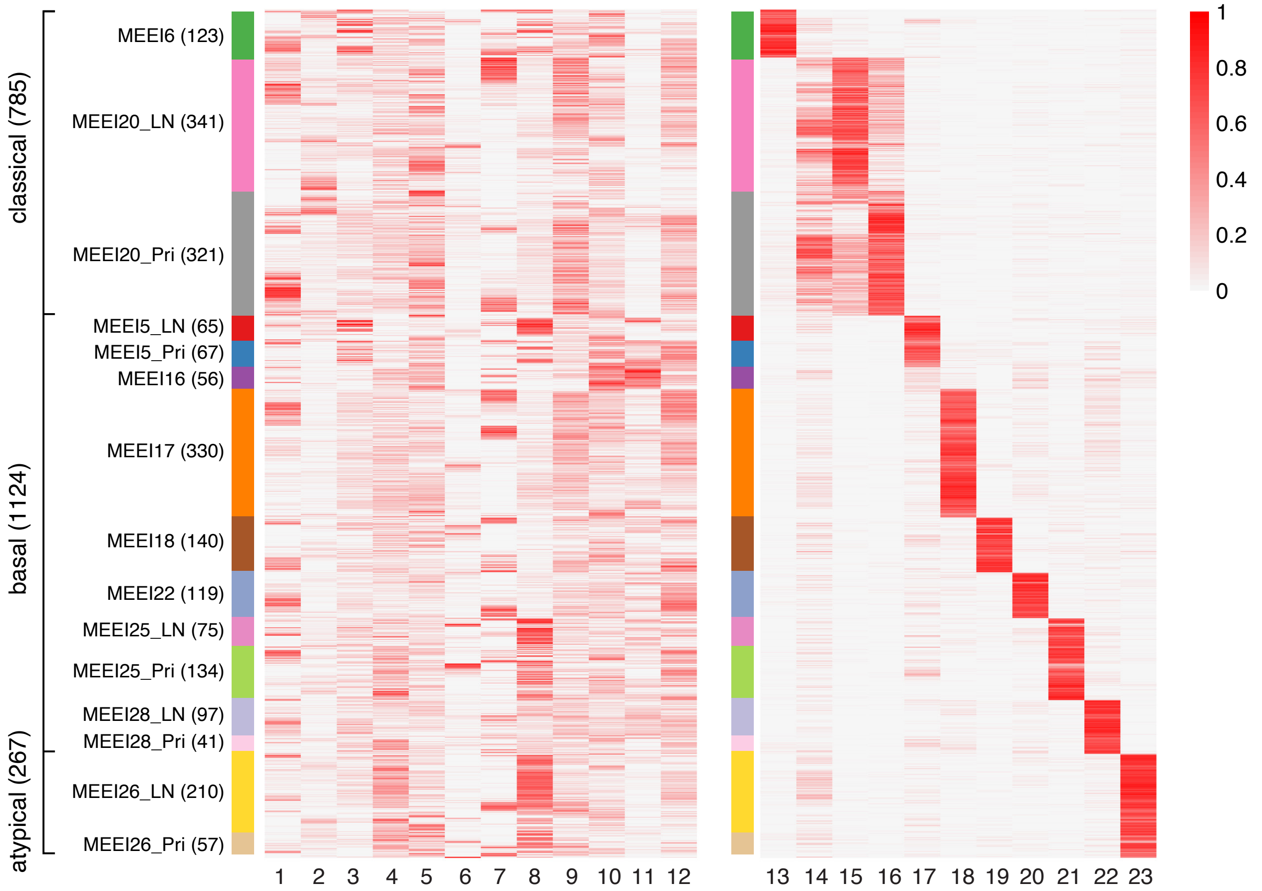

Figure S4: Result of applying NMF to the HNSCC log-pc count data. The heatmap shows the membership estimates for the 2,176 cells (rows) and the 23 GEPs identified by NMF (columns). Cells are arranged top-to-bottom by tumor molecular subtype and patient, and GEPs are grouped left-to-right based on whether they are more patient-specific (GEP 13–23) or more shared across patients (GEP 1–12). For the heatmap, membership values were rescaled separately for each GEP so that the maximum membership for each GEP was always 1.

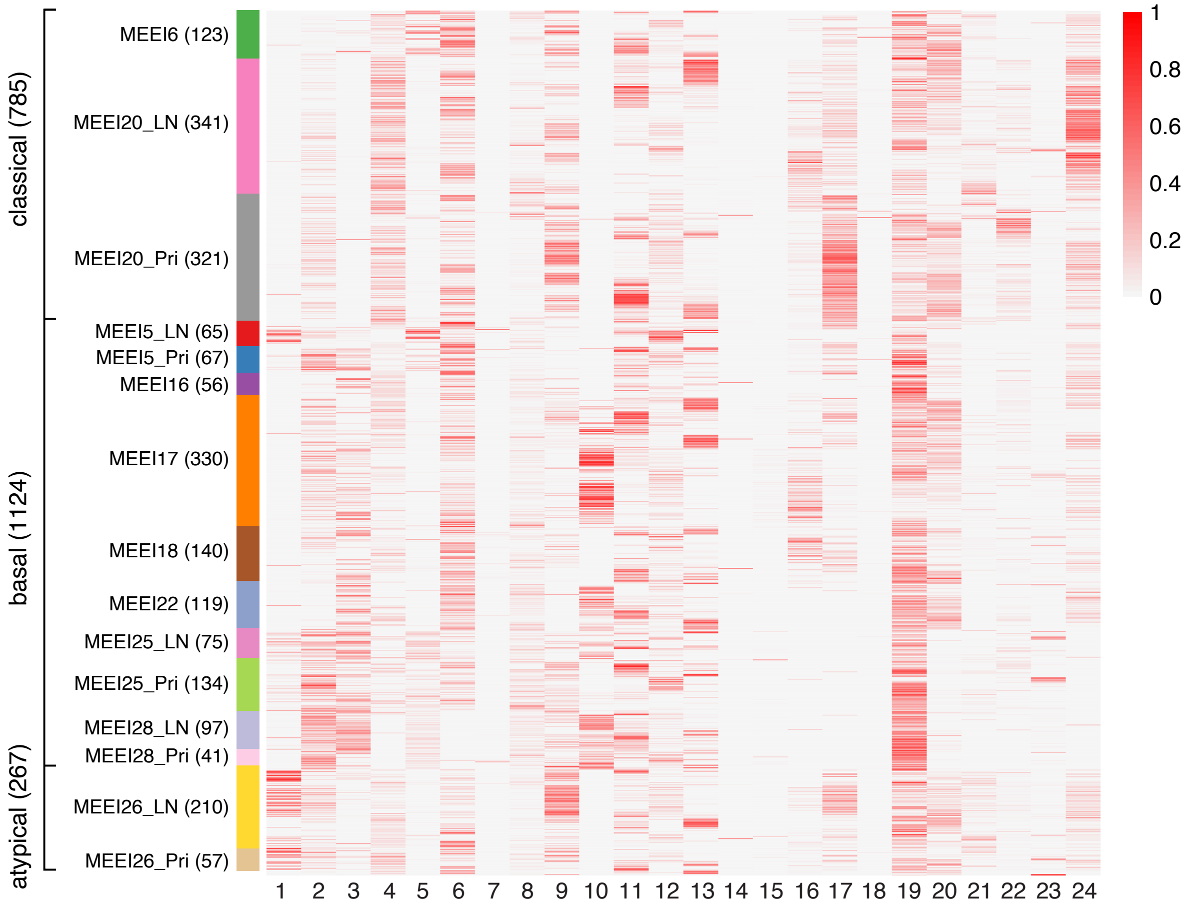

Figure S5: Result of applying LIGER to the HNSCC log-pc count data. The heatmap shows the membership estimates for the 2,176 cells (rows) and the 24 GEPs identified by LIGER (columns). Cells are arranged top-to-bottom by tumor molecular subtype and patient. Membership values were rescaled separately for each GEP so that the maximum membership for each GEP was always 1.

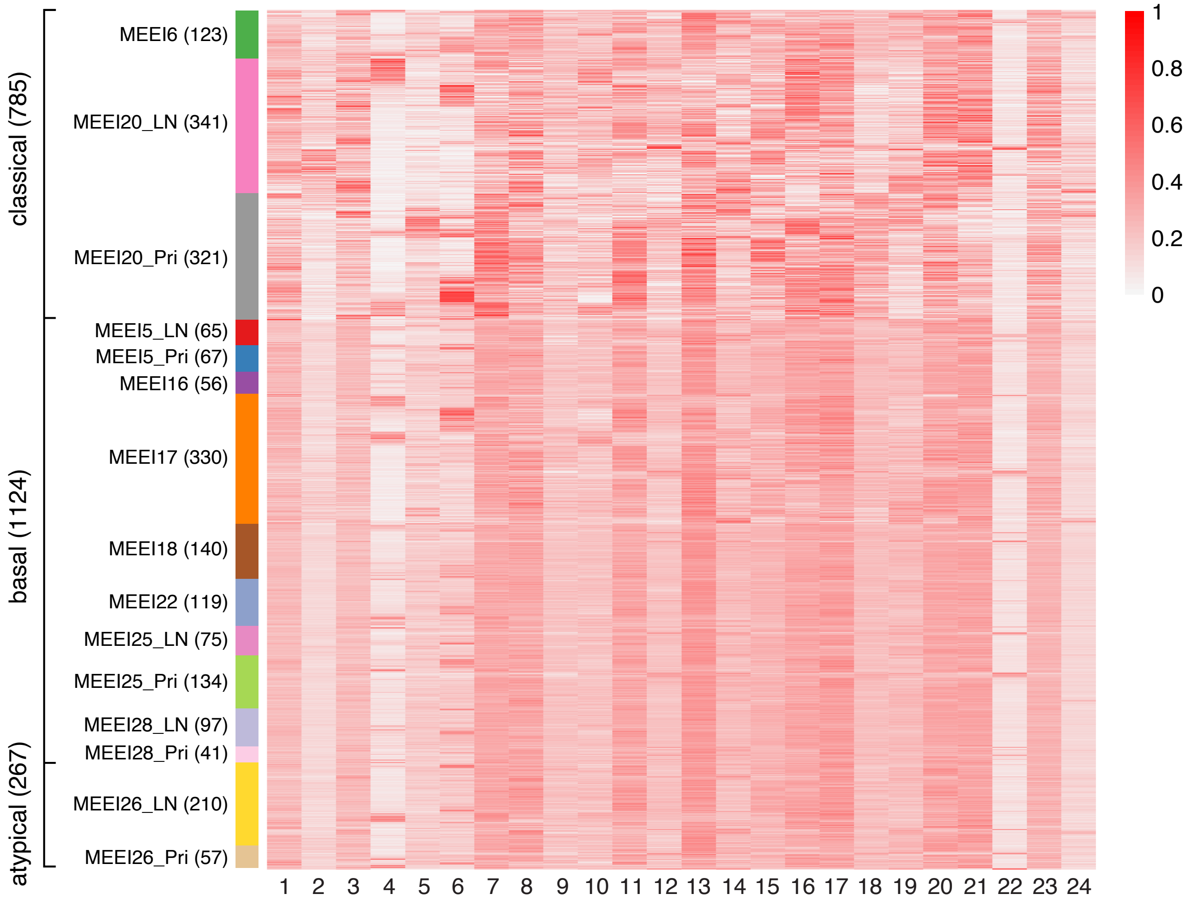

Figure S6: Result of applying CCA (implemented in Seurat) to the HNSCC log-pc count data. The heatmap shows the membership estimates for the 2,176 cells (rows) and the 24 GEPs identified by CCA (columns). Cells are arranged top-to-bottom by tumor molecular subtype and patient. Membership values were rescaled separately for each GEP so that the maximum membership for each GEP was always 1.

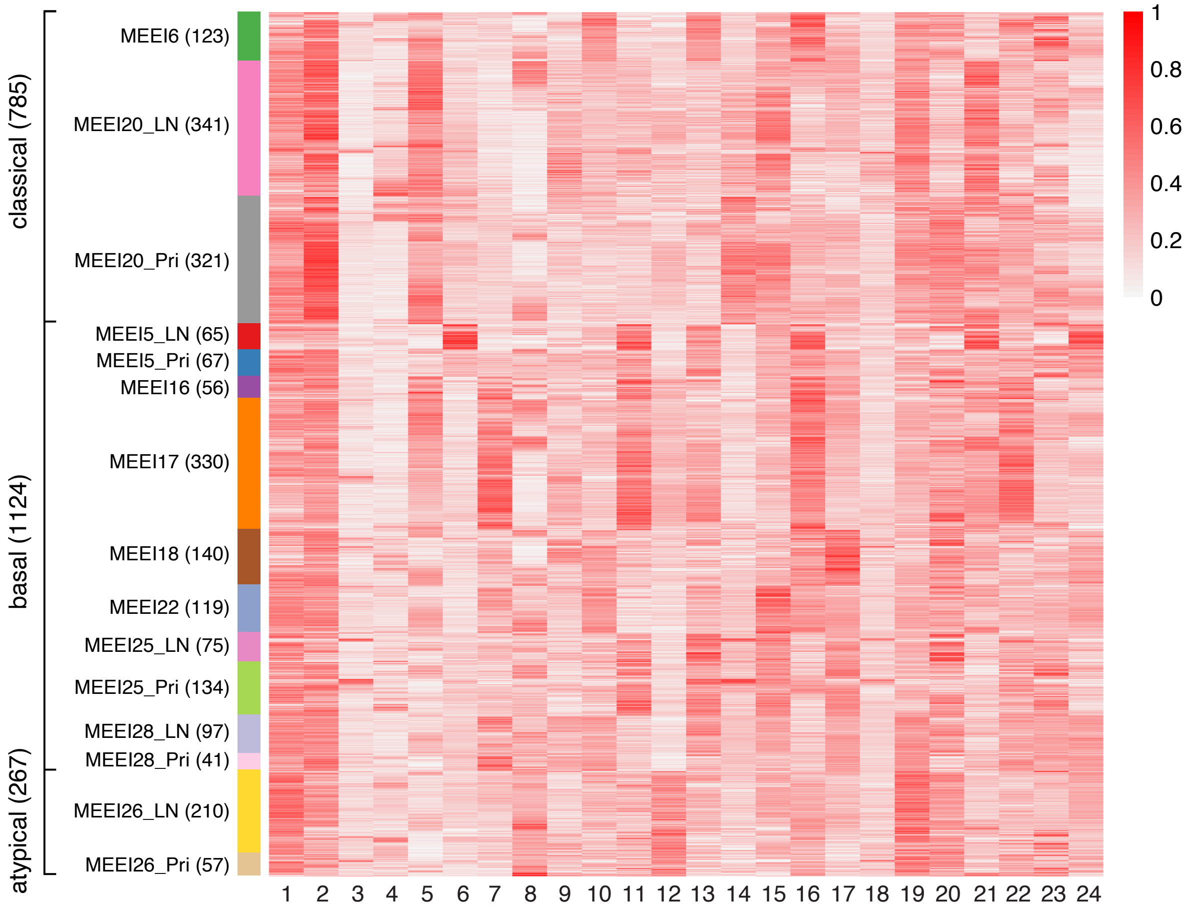

Figure S7: Result of applying MNN Correct to the HNSCC log-pc count data. The heatmap shows the membership estimates for the 2,176 cells (rows) and the 24 GEPs identified by MNN Correct (columns). Cells are arranged top-to-bottom by tumor molecular subtype and patient. Membership values were rescaled separately for each GEP so that the maximum membership for each GEP was always 1.

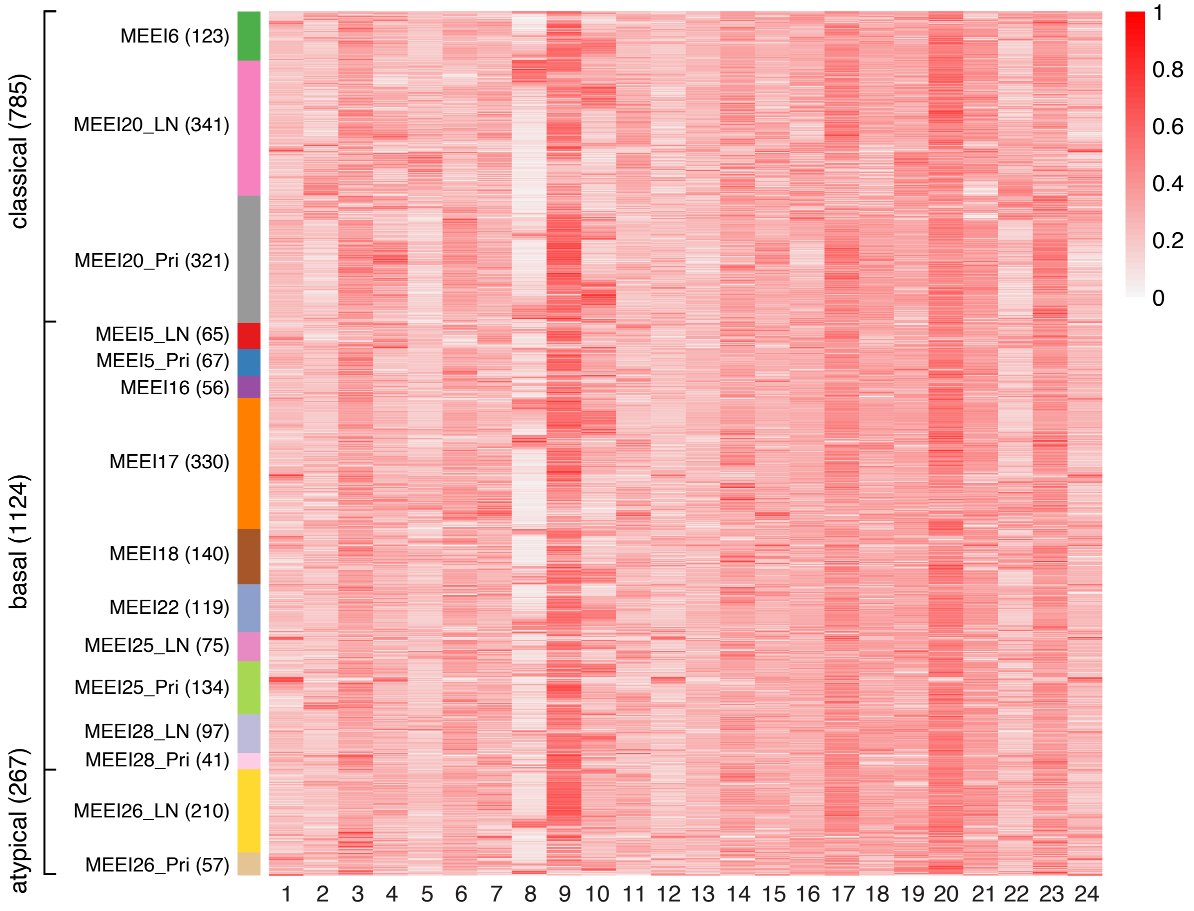

Figure S8: Result of applying Conos to the HNSCC log-pc count data. The heatmap shows the membership estimates for the 2,176 cells (rows) and the 24 GEPs identified by Conos (columns). Cells are arranged top-to-bottom by tumor molecular subtype and patient. Membership values were rescaled separately for each GEP so that the maximum membership for each GEP was always 1.

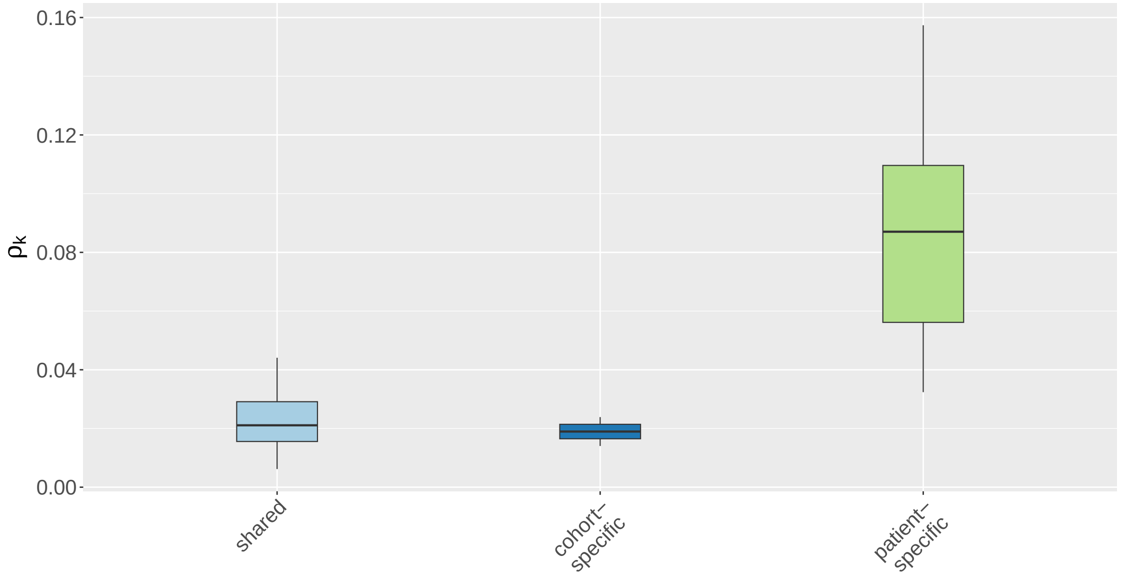

Figure S9: Boxplots of ρ_k, which quantifies spatial structure of GEP signatures, for GEP $k = 1, \ldots, 34$ in the GBCD fit to the PDAC data, separately for shared (1-14), cohort-specific (15-16) and patient-specific (17-34) GEPs.

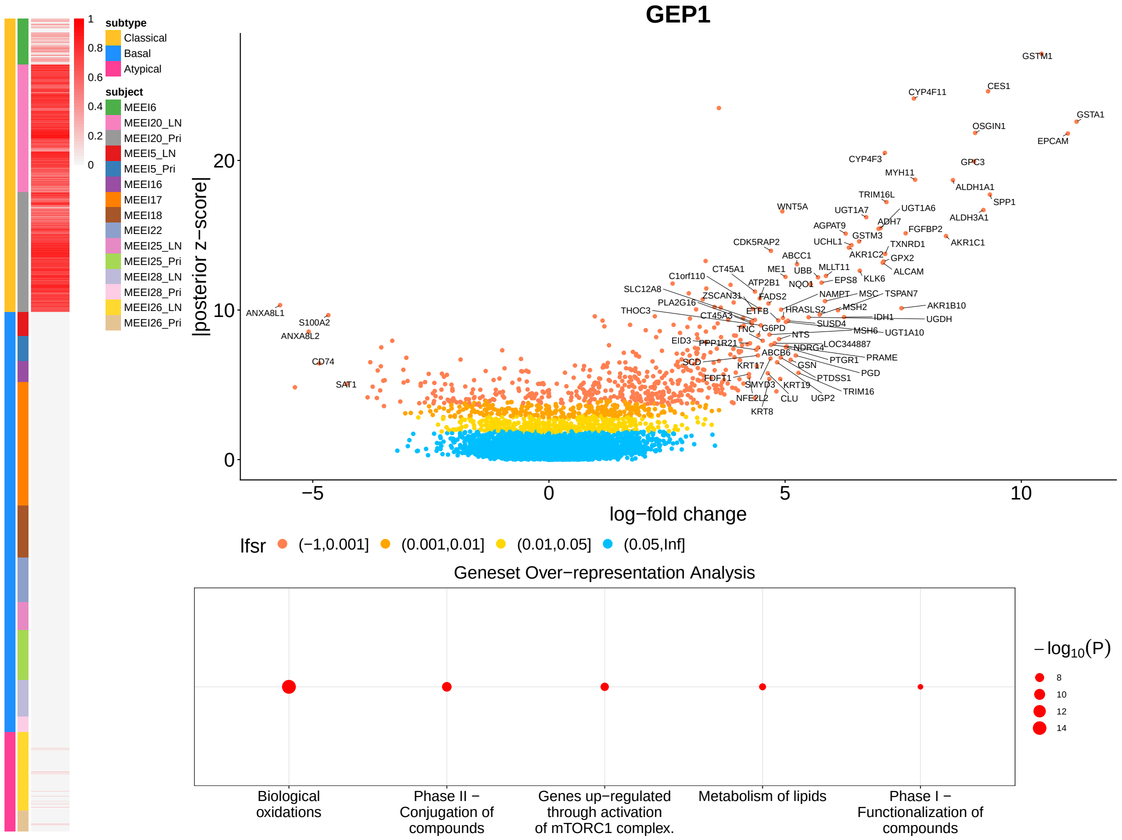
GEP1
subtype
Classical
Basal
Atypical
subject
MEEI6
MEEI20_LN
MEEI20_Pri
MEEI5_LN
MEEI5_Pri
MEEI16
MEEI17
MEEI18
MEEI22
MEEI25_LN
MEEI25_Pri
MEEI28_LN
MEEI28_Pri
MEEI26_LN
MEEI26_Pri
1
0.8
0.6
0.4
0.2
0
|posterior z−score|
log−fold change
lfsr (−1,0.001] (0.001,0.01] (0.01,0.05] (0.05,Inf]
GSTM1
CYP4F11
CES1
GSTA1
OSGIN1
EPCAM
CYP4F3
GPC3
MYH11
ALDH1A1
SPP1
TRIM16L
WNT5A
UGT1A7
UGT1A6
ALDH3A1
AGPAT9
ADH7
FGFBP2
GSTM3
UCHL1
TXNRD1
AKR1C1
CDK5RAP2
ABCC1
AKR1C2
GPX2
CT45A1
ME1
UBB
MLLT11
ALCAM
C1orf110
ATP2B1
NQO1
EPS8
KLK6
SLC12A8
ZSCAN31
NAMPT
MSC
TSPAN7
PLA2G16
FADS2
AKR1B10
THOC3
ETFB
HRASLS2
MSH2
CT45A3
IDH1
UGDH
ANXA8L1
S100A2
TNC
G6PD
SUSD4
ANXA8L2
NTS
MSH6
UGT1A10
EID3
PPP1R21
NDRG4
LOC344887
CD74
ABCB6
PRAME
SCD
KRT17
GSN
PTGR1
PGD
SAT1
FDFT1
PTDSS1
SMYD3
KRT19
TRIM16
NFE2L2
CLU
UGP2
KRT8
Geneset Over−representation Analysis
Biological oxidations
Phase II − Conjugation of compounds
Genes up−regulated through activation of mTORC1 complex.
Metabolism of lipids
Phase I − Functionalization of compounds
$-\log_{10}(P)$
8
10
12
14

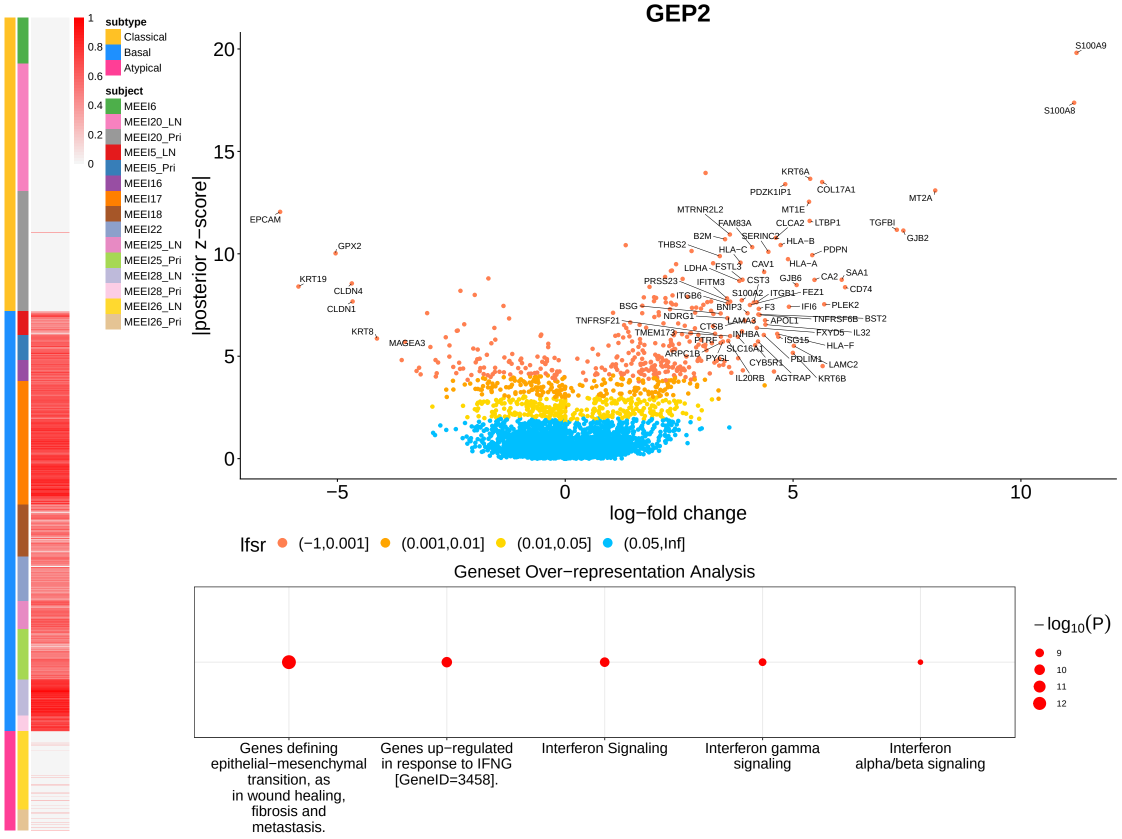
GEP2
subtype
Classical
Basal
Atypical
subject
MEEI6
MEEI20_LN
MEEI20_Pri
MEEI5_LN
MEEI5_Pri
MEEI16
MEEI17
MEEI18
MEEI22
MEEI25_LN
MEEI25_Pri
MEEI28_LN
MEEI28_Pri
MEEI26_LN
MEEI26_Pri
1
0.8
0.6
0.4
0.2
0
|posterior z−score|
log−fold change
lfsr (−1,0.001] (0.001,0.01] (0.01,0.05] (0.05,Inf]
S100A9
S100A8
KRT6A
PDZK1IP1
COL17A1
MT2A
MTRNR2L2
MT1E
EPCAM
FAM83A
CLCA2
LTBP1
TGFBI
GJB2
B2M
SERINC2
HLA−B
GPX2
THBS2
HLA−C
PDPN
CAV1
HLA−A
LDHA
FSTL3
KRT19
PRSS23
IFITM3
CST3
GJB6
CA2
SAA1
CLDN4
CD74
ITGB6
S100A2
ITGB1
FEZ1
CLDN1
BSG
BNIP3
F3
IFI6
PLEK2
NDRG1
TNFRSF21
LAMA3
APOL1
TNFRSF6B
BST2
KRT8
CTSB
TMEM173
INHBA
FXYD5
IL32
MAGEA3
PTRF
ISG15
SLC16A1
HLA−F
ARPC1B
PYGL
PDLIM1
CYB5R1
LAMC2
IL20RB
AGTRAP
KRT6B
Geneset Over−representation Analysis
Genes defining epithelial−mesenchymal transition, as in wound healing, fibrosis and metastasis.
Genes up−regulated in response to IFNG [GeneID=3458].
Interferon Signaling
Interferon gamma signaling
Interferon alpha/beta signaling
$-\log_{10}(P)$
9
10
11
12

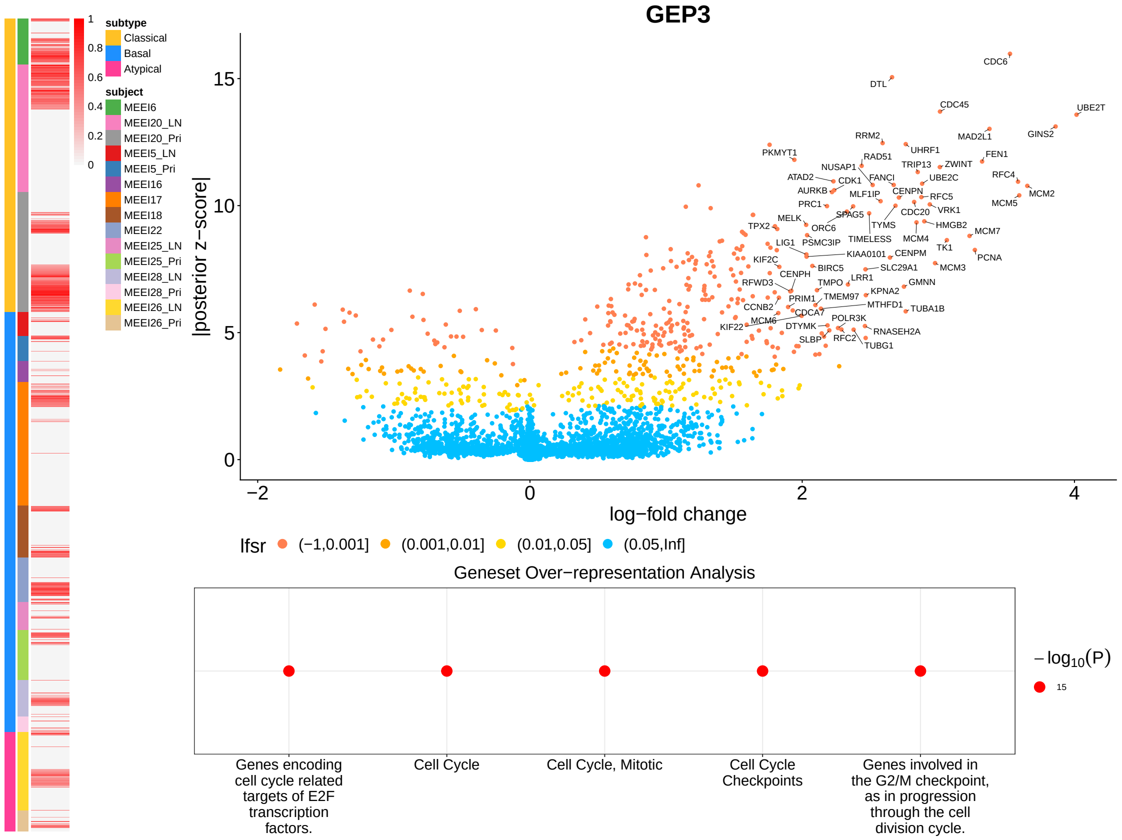
GEP3
subtype
Classical
Basal
Atypical
subject
MEEI6
MEEI20_LN
MEEI20_Pri
MEEI5_LN
MEEI5_Pri
MEEI16
MEEI17
MEEI18
MEEI22
MEEI25_LN
MEEI25_Pri
MEEI28_LN
MEEI28_Pri
MEEI26_LN
MEEI26_Pri
|posterior z−score|
log−fold change
lfsr (−1,0.001] (0.001,0.01] (0.01,0.05] (0.05,Inf]
Geneset Over−representation Analysis
−log10(P)
15
Genes encoding cell cycle related targets of E2F transcription factors.
Cell Cycle
Cell Cycle, Mitotic
Cell Cycle Checkpoints
Genes involved in the G2/M checkpoint, as in progression through the cell division cycle.

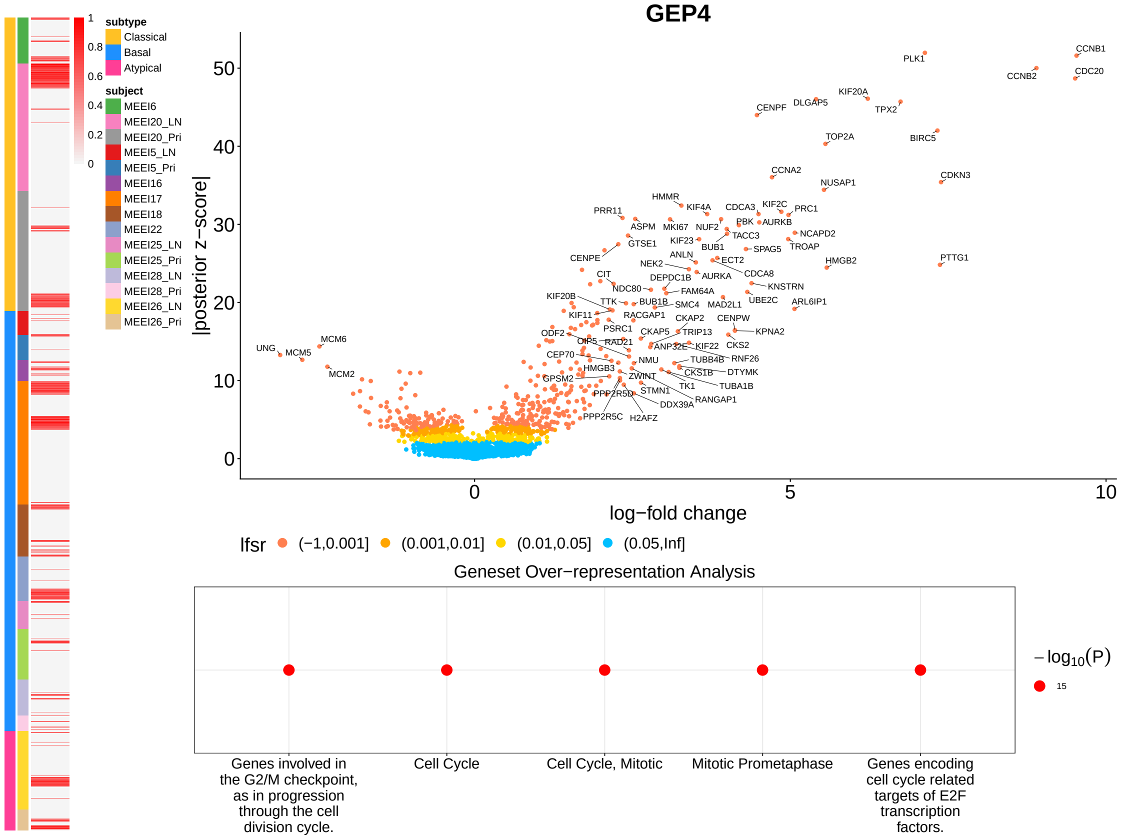
GEP4
subtype
Classical
Basal
Atypical
subject
MEEI6
MEEI20_LN
MEEI20_Pri
MEEI5_LN
MEEI5_Pri
MEEI16
MEEI17
MEEI18
MEEI22
MEEI25_LN
MEEI25_Pri
MEEI28_LN
MEEI28_Pri
MEEI26_LN
MEEI26_Pri
|posterior z−score|
log−fold change
lfsr (−1,0.001] (0.001,0.01] (0.01,0.05] (0.05,Inf]
Geneset Over−representation Analysis
−log10(P)
15
Genes involved in the G2/M checkpoint, as in progression through the cell division cycle.
Cell Cycle
Cell Cycle, Mitotic
Mitotic Prometaphase
Genes encoding cell cycle related targets of E2F transcription factors.

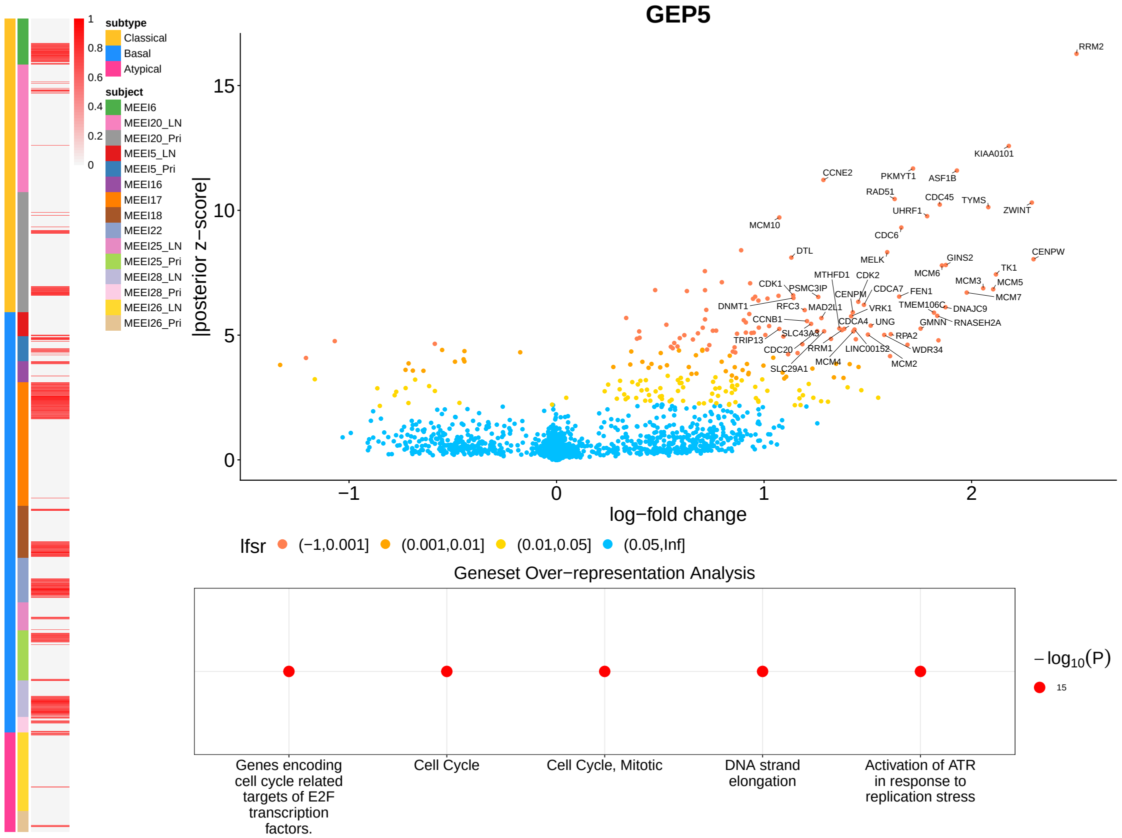
subtype
Classical
Basal
Atypical
subject
MEEI6
MEEI20_LN
MEEI20_Pri
MEEI5_LN
MEEI5_Pri
MEEI16
MEEI17
MEEI18
MEEI22
MEEI25_LN
MEEI25_Pri
MEEI28_LN
MEEI28_Pri
MEEI26_LN
MEEI26_Pri
GEP5
|posterior z−score|
log−fold change
RRM2
KIAA0101
CCNE2
PKMYT1
ASF1B
RAD51
CDC45
TYMS
ZWINT
UHRF1
MCM10
CDC6
DTL
MELK
GINS2
CENPW
TK1
MCM6
MCM3
MCM5
MCM7
CDK1
MTHFD1
CDK2
PSMC3IP
CENPM
CDCA7
FEN1
DNMT1
RFC3
MAD2L1
VRK1
TMEM106C
DNAJC9
CCNB1
CDCA4
UNG
GMNN
RNASEH2A
TRIP13
SLC43A3
RPA2
CDC20
RRM1
LINC00152
WDR34
MCM4
MCM2
SLC29A1
lfsr
(−1,0.001]
(0.001,0.01]
(0.01,0.05]
(0.05,Inf]
Geneset Over−representation Analysis
Genes encoding cell cycle related targets of E2F transcription factors.
Cell Cycle
Cell Cycle, Mitotic
DNA strand elongation
Activation of ATR in response to replication stress
$-\log_{10}(P)$
15

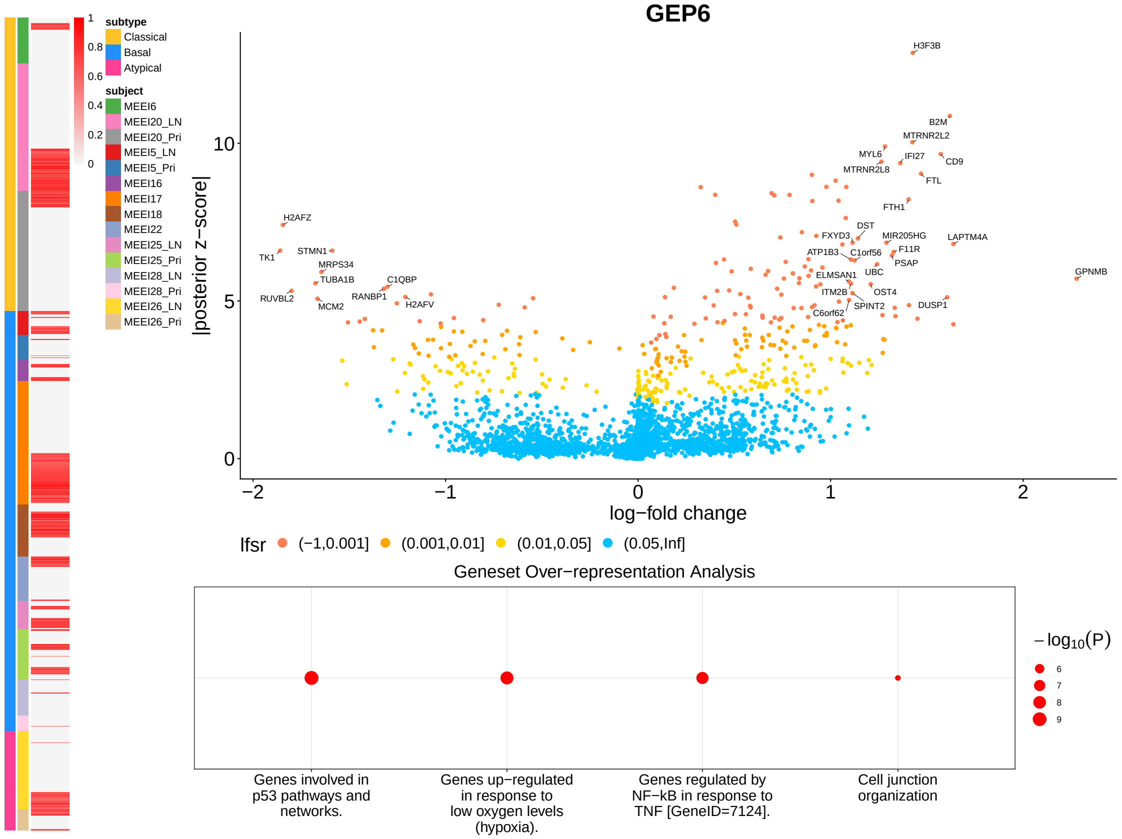
subtype
Classical
Basal
Atypical
subject
MEEI6
MEEI20_LN
MEEI20_Pri
MEEI5_LN
MEEI5_Pri
MEEI16
MEEI17
MEEI18
MEEI22
MEEI25_LN
MEEI25_Pri
MEEI28_LN
MEEI28_Pri
MEEI26_LN
MEEI26_Pri
GEP6
|posterior z−score|
log−fold change
H3F3B
B2M
MTRNR2L2
MYL6
IFI27
CD9
MTRNR2L8
FTL
FTH1
H2AFZ
DST
FXYD3
MIR205HG
LAPTM4A
TK1
STMN1
ATP1B3
C1orf56
F11R
MRPS34
PSAP
TUBA1B
C1QBP
ELMSAN1
UBC
GPNMB
RUVBL2
RANBP1
ITM2B
OST4
MCM2
H2AFV
SPINT2
DUSP1
C6orf62
lfsr
(−1,0.001]
(0.001,0.01]
(0.01,0.05]
(0.05,Inf]
Geneset Over−representation Analysis
Genes involved in p53 pathways and networks.
Genes up−regulated in response to low oxygen levels (hypoxia).
Genes regulated by NF−kB in response to TNF [GeneID=7124].
Cell junction organization
$-\log_{10}(P)$
6
7
8
9

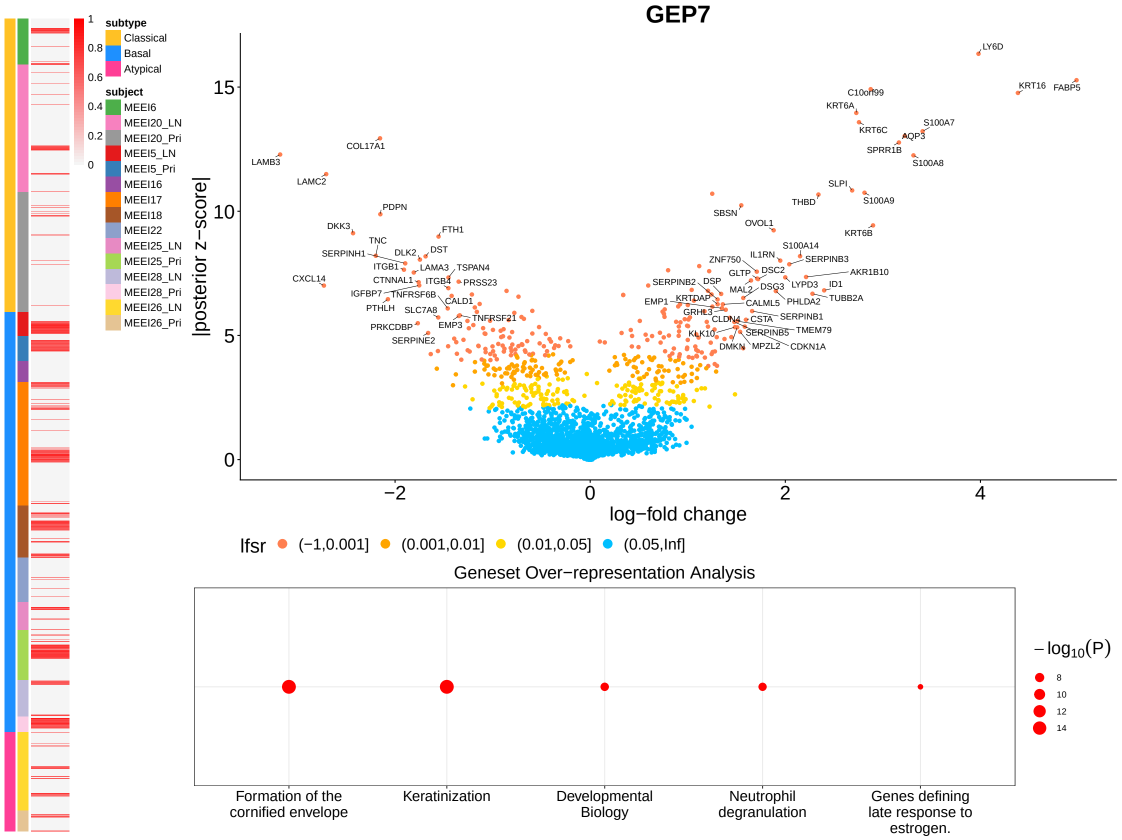
GEP7
subtype
Classical
Basal
Atypical
subject
MEEI6
MEEI20_LN
MEEI20_Pri
MEEI5_LN
MEEI5_Pri
MEEI16
MEEI17
MEEI18
MEEI22
MEEI25_LN
MEEI25_Pri
MEEI28_LN
MEEI28_Pri
MEEI26_LN
MEEI26_Pri
1
0.8
0.6
0.4
0.2
0
|posterior z−score|
log−fold change
lfsr
(−1,0.001]
(0.001,0.01]
(0.01,0.05]
(0.05,Inf]
LY6D
KRT16
FABP5
C10orf99
KRT6A
S100A7
KRT6C
AQP3
SPRR1B
S100A8
COL17A1
LAMB3
LAMC2
SLPI
THBD
S100A9
SBSN
OVOL1
KRT6B
PDPN
DKK3
FTH1
TNC
DST
SERPINH1
DLK2
ITGB1
LAMA3
TSPAN4
CTNNAL1
ITGB4
PRSS23
CXCL14
IGFBP7
TNFRSF6B
CALD1
PTHLH
SLC7A8
TNFRSF21
PRKCDBP
EMP3
SERPINE2
S100A14
IL1RN
ZNF750
SERPINB3
GLTP
DSC2
AKR1B10
SERPINB2
DSP
LYPD3
ID1
MAL2
DSG3
TUBB2A
EMP1
KRTDAP
CALML5
PHLDA2
GRHL3
CLDN4
CSTA
SERPINB1
KLK10
SERPINB5
TMEM79
DMKN
MPZL2
CDKN1A
Geneset Over−representation Analysis
Formation of the cornified envelope
Keratinization
Developmental Biology
Neutrophil degranulation
Genes defining late response to estrogen.
−log10(P)
8
10
12
14

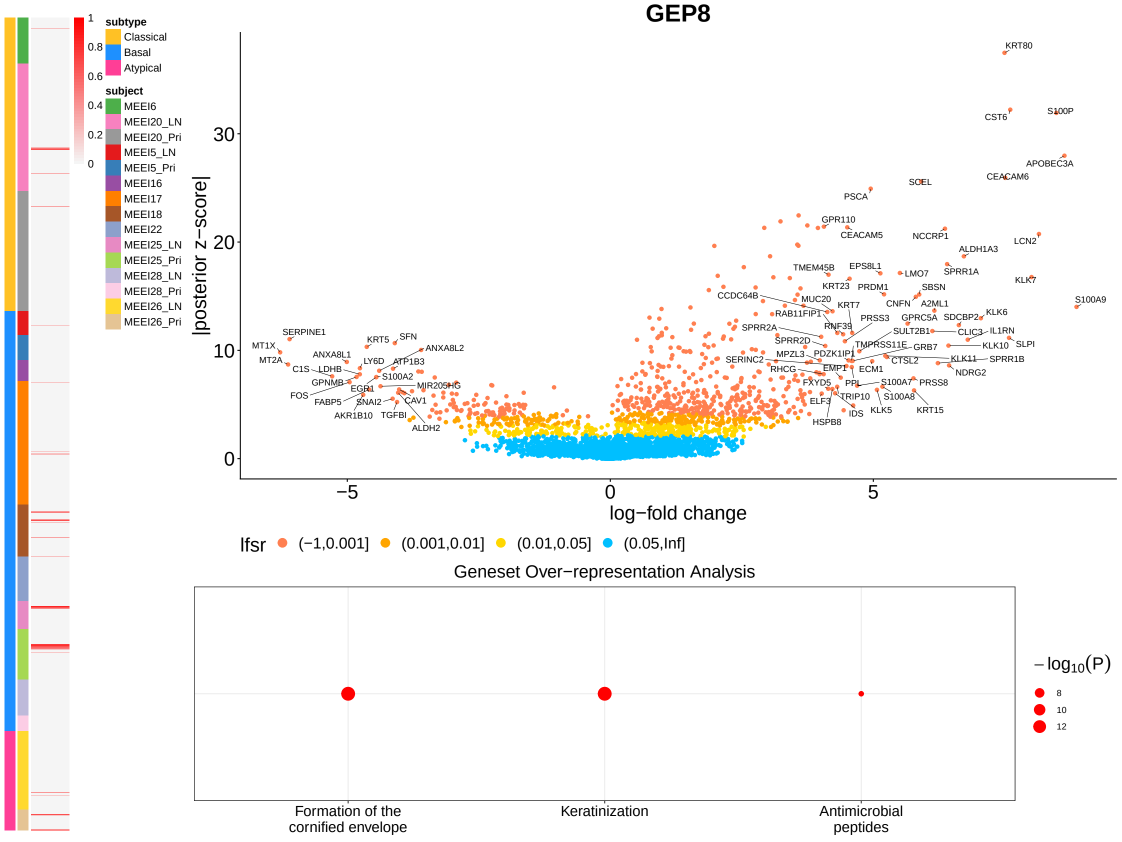
GEP8
subtype
Classical
Basal
Atypical
subject
MEEI6
MEEI20_LN
MEEI20_Pri
MEEI5_LN
MEEI5_Pri
MEEI16
MEEI17
MEEI18
MEEI22
MEEI25_LN
MEEI25_Pri
MEEI28_LN
MEEI28_Pri
MEEI26_LN
MEEI26_Pri
1
0.8
0.6
0.4
0.2
0
|posterior z−score|
log−fold change
lfsr
(−1,0.001]
(0.001,0.01]
(0.01,0.05]
(0.05,Inf]
KRT80
CST6
S100P
APOBEC3A
CEACAM6
SOEL
PSCA
GPR110
CEACAM5
NCCRP1
ALDH1A3
LCN2
TMEM45B
EPS8L1
LMO7
SPRR1A
KRT23
PRDM1
SBSN
KLK7
CCDC64B
MUC20
KRT7
CNFN
A2ML1
S100A9
RAB11FIP1
PRSS3
GPRC5A
SDCBP2
KLK6
SPRR2A
RNF39
SULT2B1
CLIC3
IL1RN
SPRR2D
TMPRSS11E
GRB7
KLK10
SLPI
MPZL3
PDZK1IP1
SERINC2
CTSL2
KLK11
SPRR1B
RHCG
EMP1
ECM1
NDRG2
FXYD5
PPL
S100A7
PRSS8
ELF3
TRIP10
S100A8
IDS
KLK5
KRT15
HSPB8
SERPINE1
MT1X
KRT5
SFN
ANXA8L2
MT2A
ANXA8L1
LY6D
ATP1B3
C1S
LDHB
S100A2
GPNMB
EGR1
MIR205HG
FOS
FABP5
SNAI2
CAV1
AKR1B10
TGFBI
ALDH2
Geneset Over−representation Analysis
Formation of the cornified envelope
Keratinization
Antimicrobial peptides
−log10(P)
8
10
12

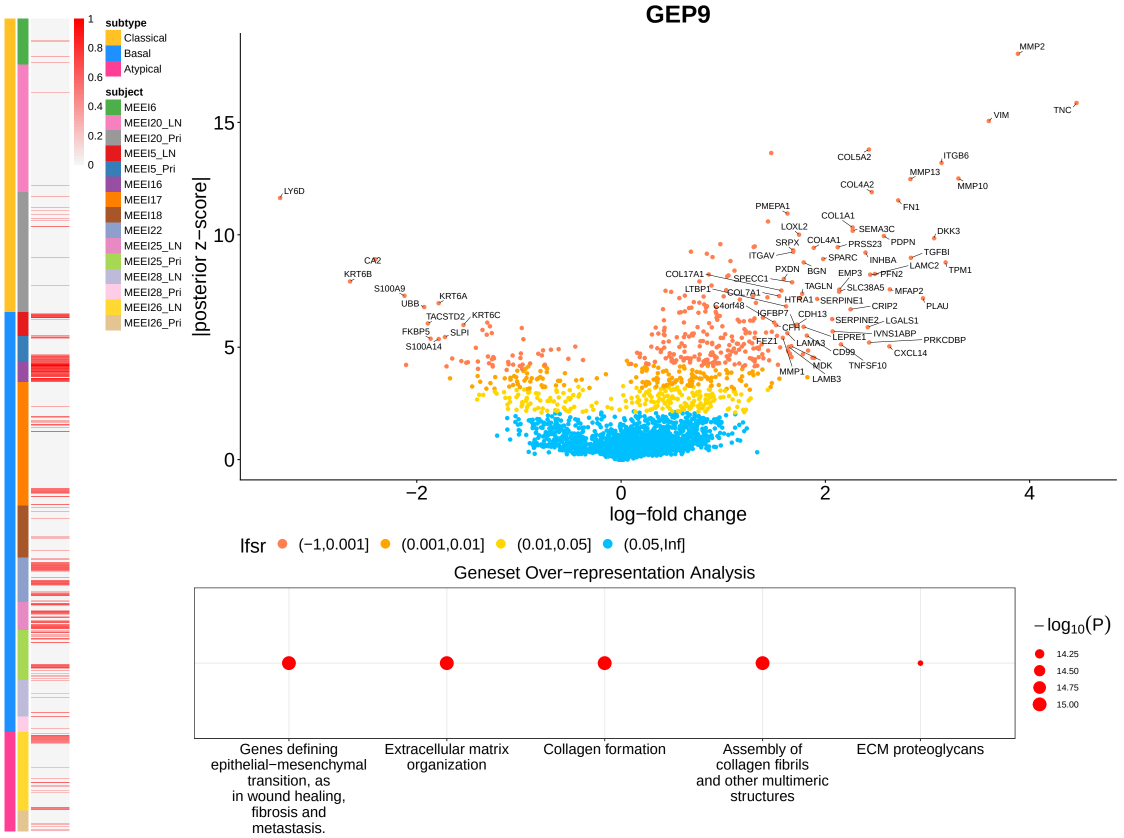

GEP9
subtype
Classical
Basal
Atypical
subject
MEEI6
MEEI20_LN
MEEI20_Pri
MEEI5_LN
MEEI5_Pri
MEEI16
MEEI17
MEEI18
MEEI22
MEEI25_LN
MEEI25_Pri
MEEI28_LN
MEEI28_Pri
MEEI26_LN
MEEI26_Pri
|posterior z−score|
log−fold change
lfsr (−1,0.001] (0.001,0.01] (0.01,0.05] (0.05,Inf]
MMP2
TNC
VIM
ITGB6
MMP13
MMP10
COL5A2
COL4A2
FN1
LY6D
PMEPA1
COL1A1
LOXL2
SEMA3C
DKK3
CA2
KRT6B
S100A9
UBB
KRT6A
TACSTD2
KRT6C
FKBP5
SLPI
S100A14
Geneset Over−representation Analysis
Genes defining epithelial−mesenchymal transition, as in wound healing, fibrosis and metastasis.
Extracellular matrix organization
Collagen formation
Assembly of collagen fibrils and other multimeric structures
ECM proteoglycans
−log10(P)
14.25
14.50
14.75
15.00

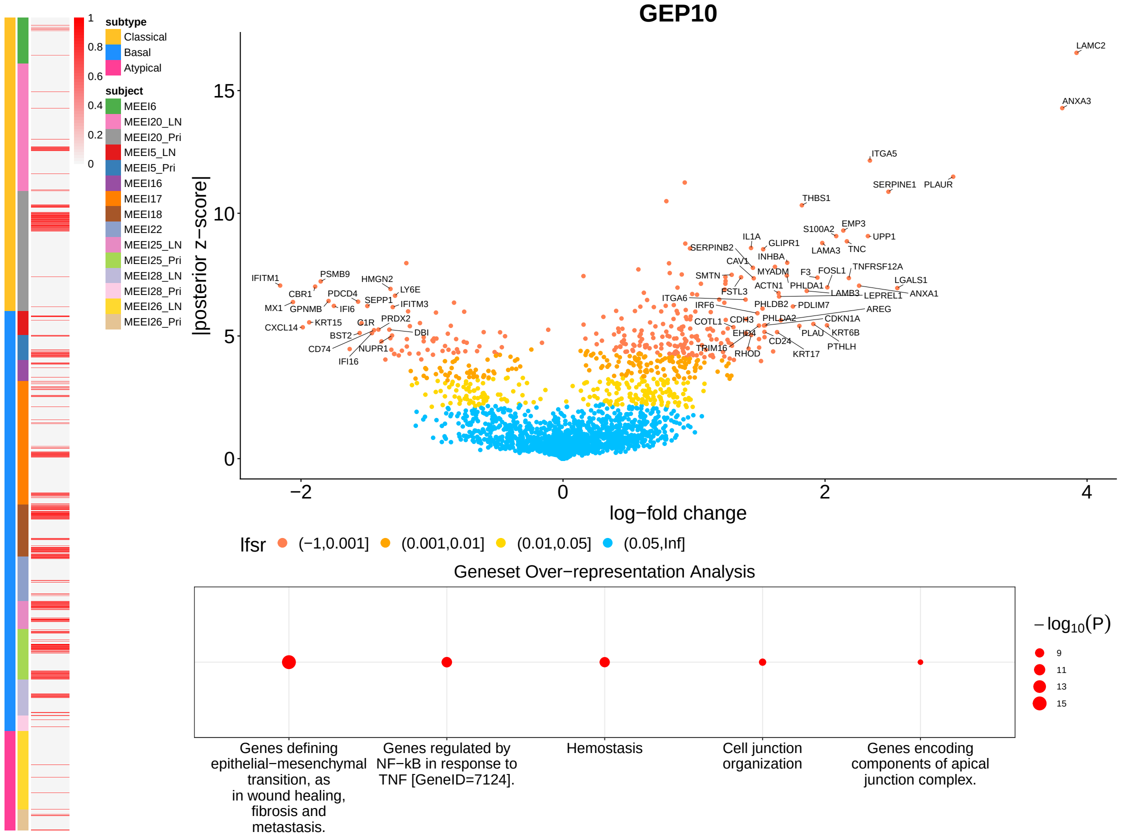

GEP10
subtype
Classical
Basal
Atypical
subject
MEEI6
MEEI20_LN
MEEI20_Pri
MEEI5_LN
MEEI5_Pri
MEEI16
MEEI17
MEEI18
MEEI22
MEEI25_LN
MEEI25_Pri
MEEI28_LN
MEEI28_Pri
MEEI26_LN
MEEI26_Pri
|posterior z−score|
log−fold change
lfsr (−1,0.001] (0.001,0.01] (0.01,0.05] (0.05,Inf]
LAMC2
ANXA3
ITGA5
SERPINE1
PLAUR
THBS1
EMP3
S100A2
UPP1
IFITM1
PSMB9
HMGN2
LY6E
CBR1
PDCD4
MX1
GPNMB
CXCL14
CD74
IFI16
Geneset Over−representation Analysis
Genes defining epithelial−mesenchymal transition, as in wound healing, fibrosis and metastasis.
Genes regulated by NF−kB in response to TNF [GeneID=7124].
Hemostasis
Cell junction organization
Genes encoding components of apical junction complex.
−log10(P)
9
11
13
15

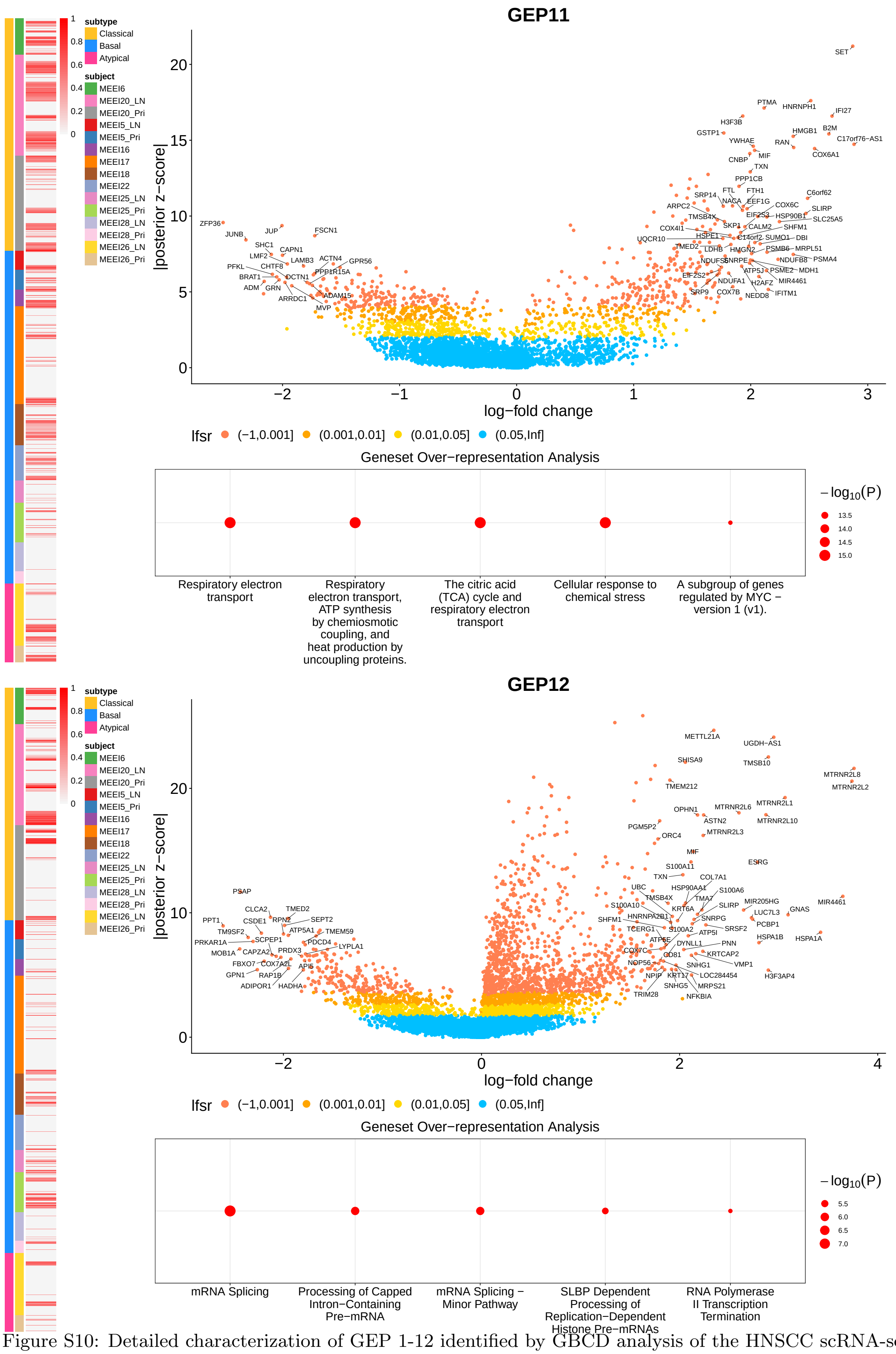

Figure S10: Detailed characterization of GEP 1-12 identified by GBCD analysis of the HNSCC scRNA-seq dataset.

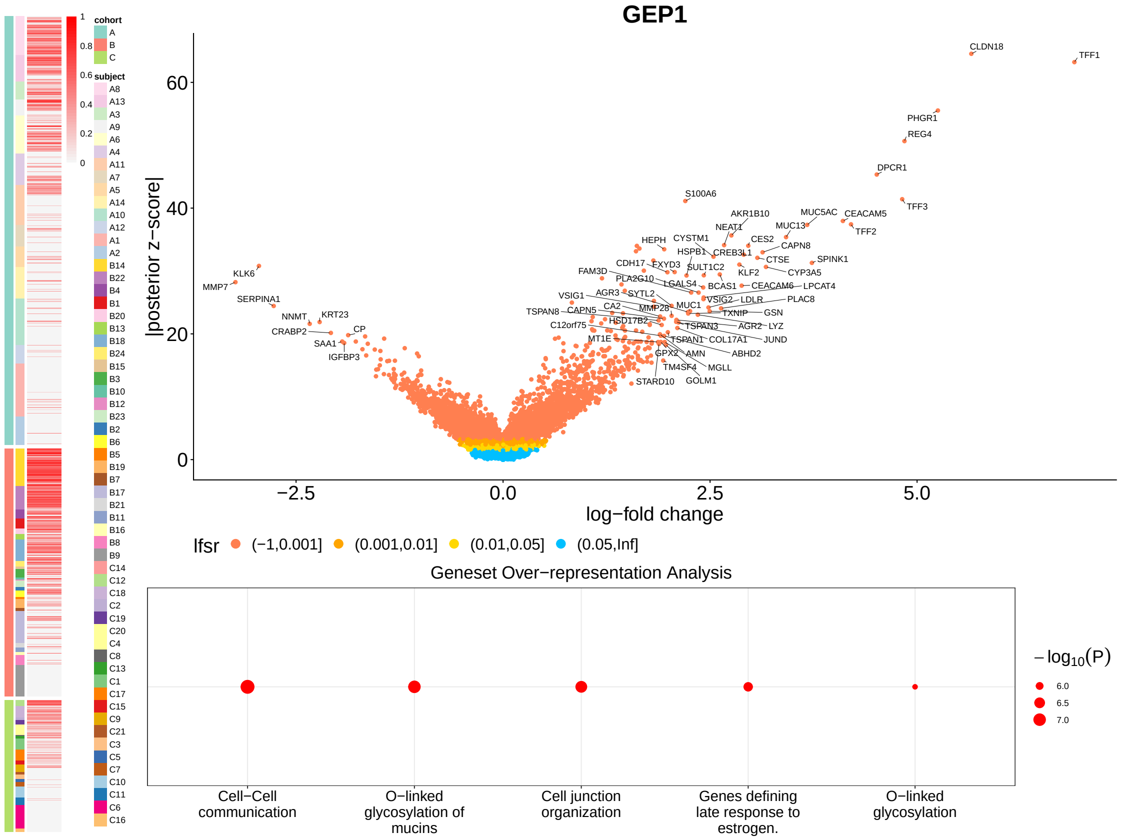
GEP1
cohort
A
B
C
subject
|posterior z−score|
log−fold change
lfsr (−1,0.001] (0.001,0.01] (0.01,0.05] (0.05,Inf]
CLDN18
TFF1
PHGR1
REG4
DPCR1
TFF3
S100A6
MUC5AC
CEACAM5
TFF2
AKR1B10
NEAT1
MUC13
CES2
CAPN8
SPINK1
KLK6
MMP7
SERPINA1
NNMT
KRT23
CRABP2
CP
SAA1
IGFBP3
Geneset Over−representation Analysis
Cell−Cell communication
O−linked glycosylation of mucins
Cell junction organization
Genes defining late response to estrogen.
O−linked glycosylation
−log10(P)
6.0
6.5
7.0

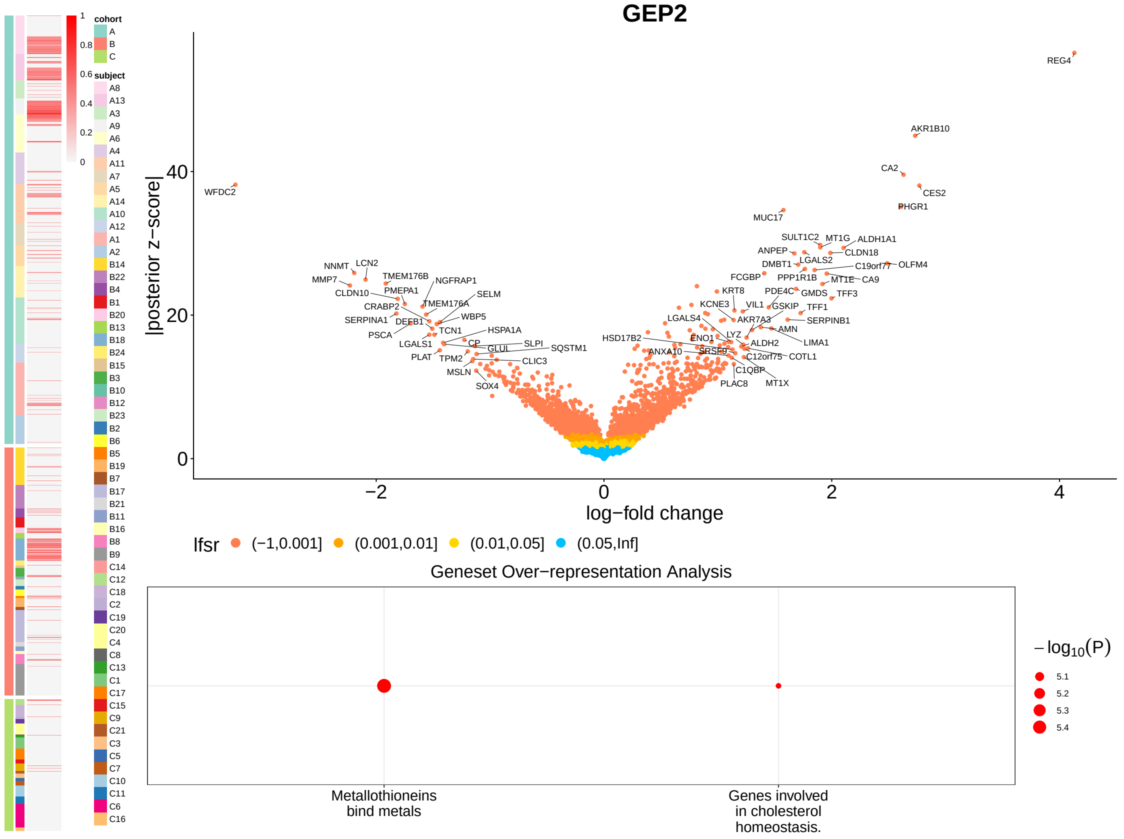
GEP2
cohort
A
B
C
subject
|posterior z−score|
log−fold change
lfsr (−1,0.001] (0.001,0.01] (0.01,0.05] (0.05,Inf]
REG4
AKR1B10
CA2
CES2
PHGR1
WFDC2
MUC17
Geneset Over−representation Analysis
Metallothioneins bind metals
Genes involved in cholesterol homeostasis.
−log10(P)
5.1
5.2
5.3
5.4

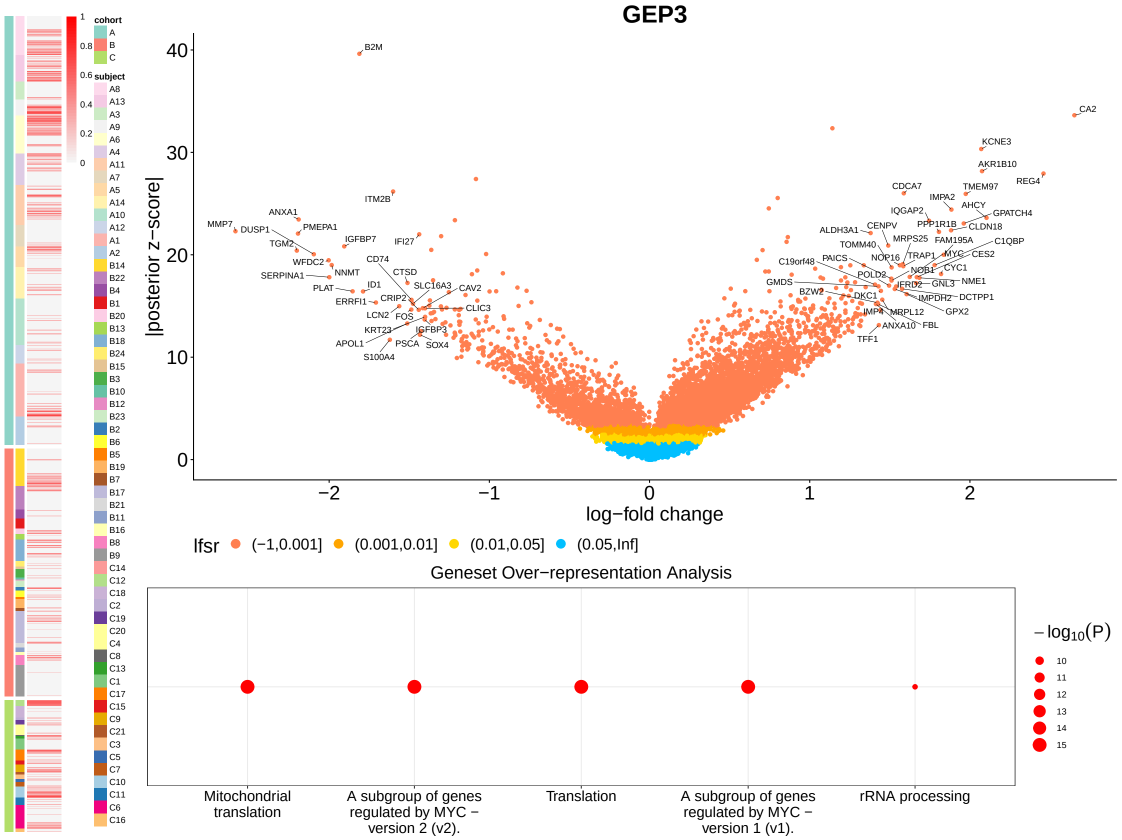

GEP3
cohort
A
B
C
subject
|posterior z−score|
log−fold change
B2M
CA2
KCNE3
AKR1B10
REG4
ITM2B
MMP7
ANXA1
DUSP1
PMEPA1
TGM2
IGFBP7
IFI27
WFDC2
SERPINA1
NNMT
PLAT
ERRFI1
CD74
CTSD
ID1
SLC16A3
CAV2
CRIP2
LCN2
FOS
CLIC3
KRT23
IGFBP3
APOL1
PSCA
SOX4
S100A4
CDCA7
TMEM97
IMPA2
AHCY
IQGAP2
GPATCH4
CENPV
PPP1R1B
CLDN18
ALDH3A1
MRPS25
FAM195A
C1QBP
TOMM40
NOP16
TRAP1
MYC
CES2
C19orf48
PAICS
NOB1
CYC1
POLD2
GMDS
IFRD2
GNL3
NME1
BZW2
DKC1
IMPDH2
DCTPP1
IMP4
MRPL12
GPX2
ANXA10
FBL
TFF1
lfsr
(−1,0.001]
(0.001,0.01]
(0.01,0.05]
(0.05,Inf]
Geneset Over−representation Analysis
Mitochondrial translation
A subgroup of genes regulated by MYC − version 2 (v2).
Translation
A subgroup of genes regulated by MYC − version 1 (v1).
rRNA processing
−log10(P)

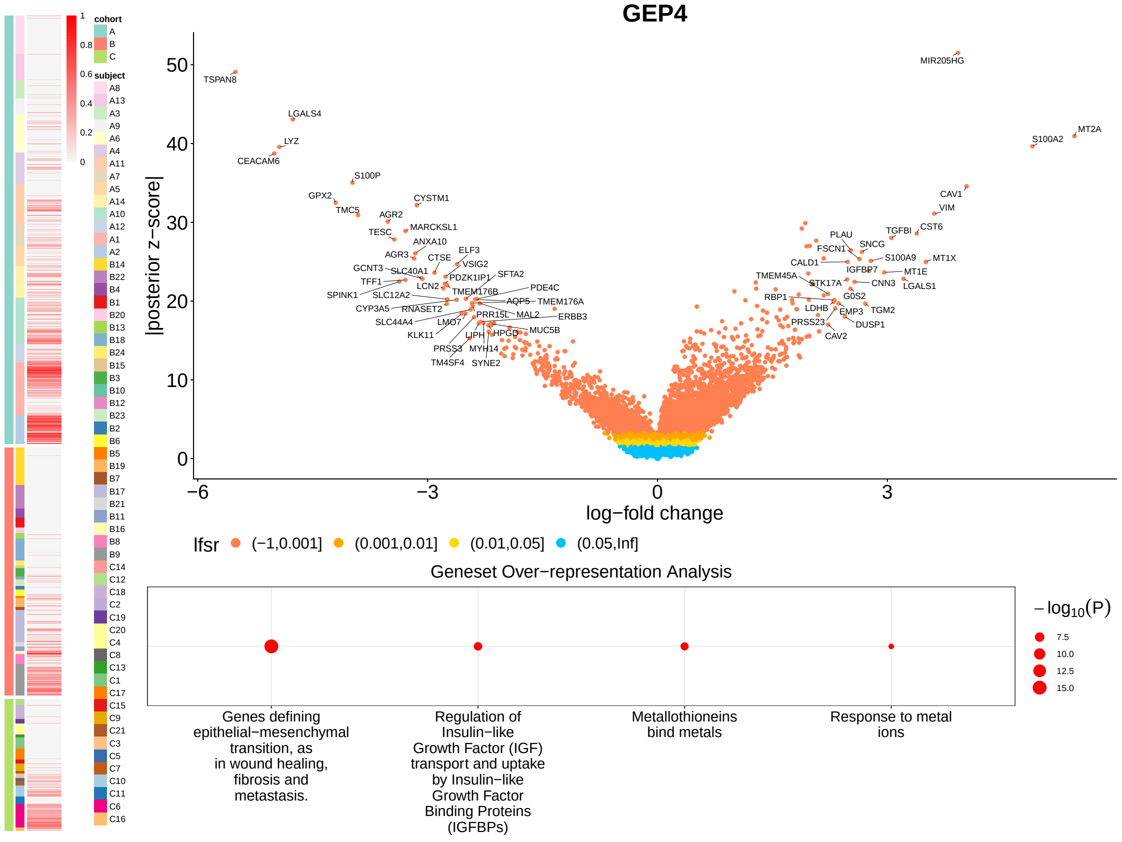

GEP4
cohort
A
B
C
subject
|posterior z−score|
log−fold change
MIR205HG
TSPAN8
LGALS4
LYZ
CEACAM6
S100A2
MT2A
S100P
GPX2
TMC5
CYSTM1
AGR2
TESC
MARCKSL1
ANXA10
AGR3
CTSE
ELF3
GCNT3
SLC40A1
VSIG2
TFF1
PDZK1IP1
SFTA2
LCN2
SPINK1
SLC12A2
TMEM176B
PDE4C
CYP3A5
RNASET2
AQP5
TMEM176A
SLC44A4
LMO7
PRR15L
MAL2
ERBB3
KLK11
LIPH
HPGD
MUC5B
PRSS3
MYH14
TM4SF4
SYNE2
CAV1
VIM
TGFBI
CST6
PLAU
FSCN1
SNCG
S100A9
MT1X
CALD1
IGFBP7
MT1E
TMEM45A
STK17A
CNN3
LGALS1
RBP1
G0S2
LDHB
EMP3
TGM2
PRSS23
DUSP1
CAV2
lfsr
(−1,0.001]
(0.001,0.01]
(0.01,0.05]
(0.05,Inf]
Geneset Over−representation Analysis
Genes defining epithelial−mesenchymal transition, as in wound healing, fibrosis and metastasis.
Regulation of Insulin−like Growth Factor (IGF) transport and uptake by Insulin−like Growth Factor Binding Proteins (IGFBPs)
Metallothioneins bind metals
Response to metal ions
−log10(P)

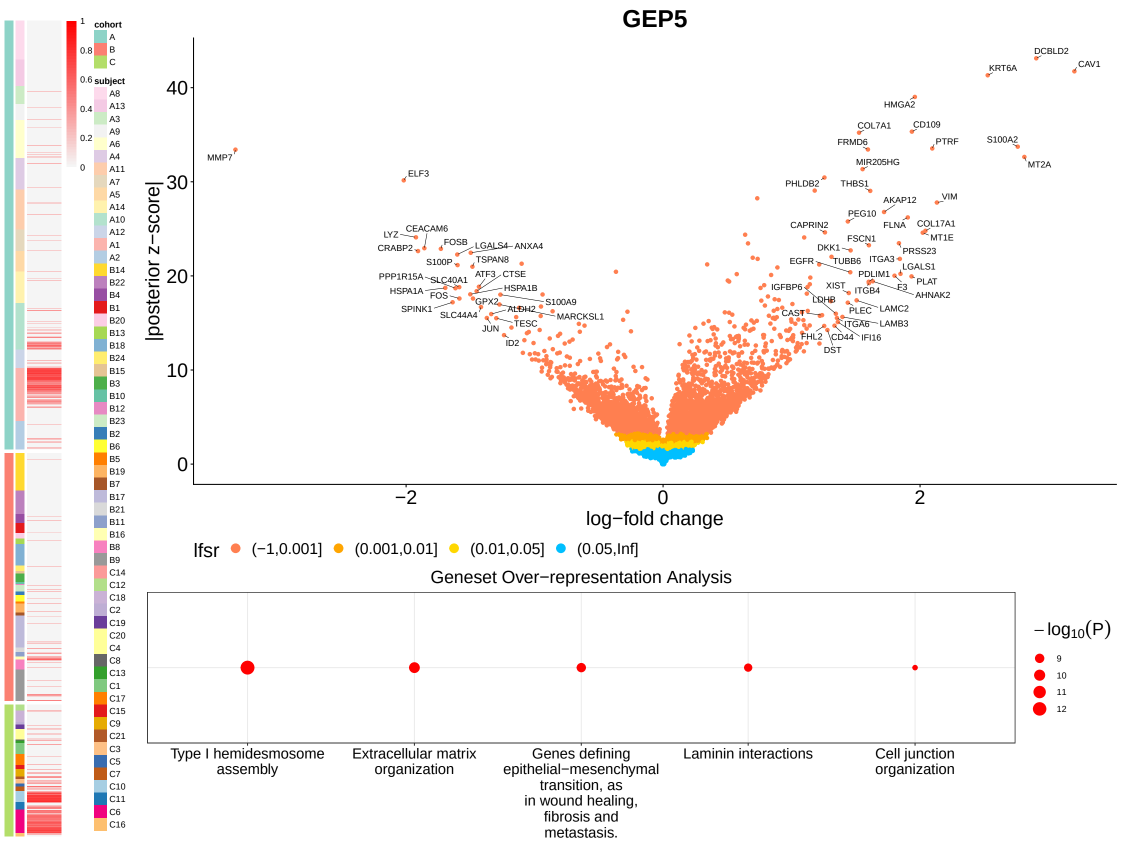
GEP5
cohort
A
B
C
subject
|posterior z−score|
log−fold change
lfsr (−1,0.001] (0.001,0.01] (0.01,0.05] (0.05,Inf]
Geneset Over−representation Analysis
Type I hemidesmosome assembly
Extracellular matrix organization
Genes defining epithelial−mesenchymal transition, as in wound healing, fibrosis and metastasis.
Laminin interactions
Cell junction organization
$-\log_{10}(P)$
9
10
11
12

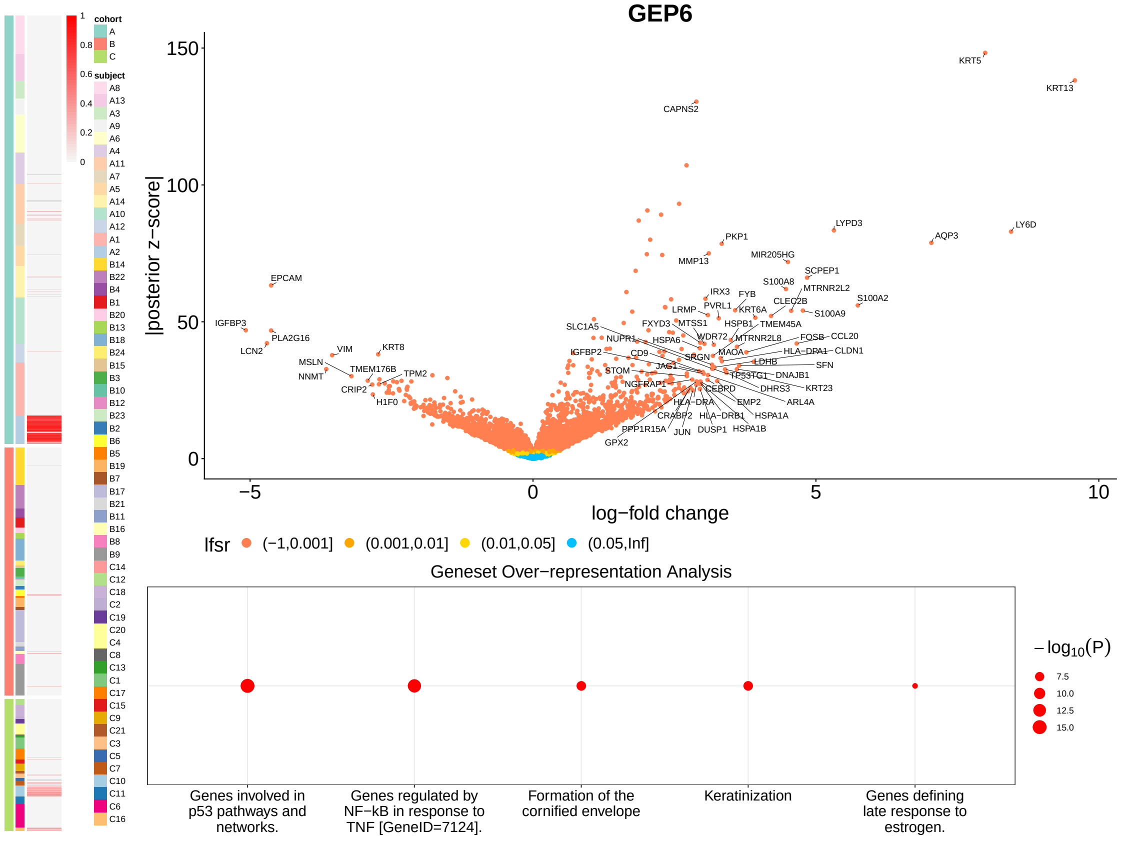
GEP6
cohort
A
B
C
subject
|posterior z−score|
log−fold change
lfsr (−1,0.001] (0.001,0.01] (0.01,0.05] (0.05,Inf]
Geneset Over−representation Analysis
Genes involved in p53 pathways and networks.
Genes regulated by NF−kB in response to TNF [GeneID=7124].
Formation of the cornified envelope
Keratinization
Genes defining late response to estrogen.
$-\log_{10}(P)$
7.5
10.0
12.5
15.0

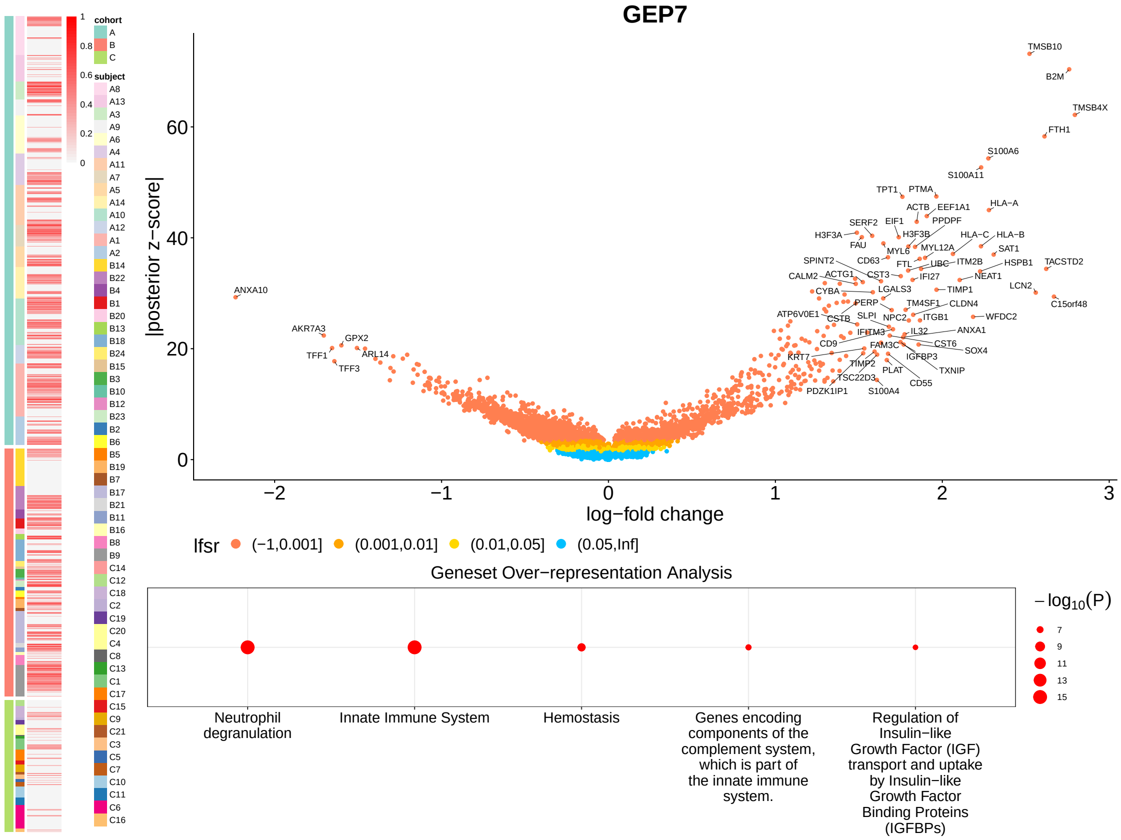
GEP7
cohort
A
B
C
subject
|posterior z−score|
log−fold change
lfsr
(−1,0.001]
(0.001,0.01]
(0.01,0.05]
(0.05,Inf]
TMSB10
B2M
TMSB4X
FTH1
S100A6
S100A11
TPT1
PTMA
ACTB
EEF1A1
HLA−A
PPDPF
SERF2
EIF1
H3F3A
H3F3B
HLA−C
HLA−B
FAU
MYL6
MYL12A
SAT1
SPINT2
CD63
FTL
UBC
ITM2B
HSPB1
TACSTD2
CALM2
ACTG1
CST3
IFI27
NEAT1
LCN2
CYBA
LGALS3
TIMP1
C15orf48
PERP
TM4SF1
CLDN4
ATP6V0E1
CSTB
SLPI
NPC2
ITGB1
WFDC2
IFITM3
IL32
ANXA1
CD9
FAM3C
CST6
SOX4
KRT7
IGFBP3
TIMP2
TXNIP
PLAT
TSC22D3
CD55
PDZK1IP1
S100A4
ANXA10
AKR7A3
GPX2
TFF1
ARL14
TFF3
Geneset Over−representation Analysis
Neutrophil degranulation
Innate Immune System
Hemostasis
Genes encoding components of the complement system, which is part of the innate immune system.
Regulation of Insulin−like Growth Factor (IGF) transport and uptake by Insulin−like Growth Factor Binding Proteins (IGFBPs)
−log10(P)

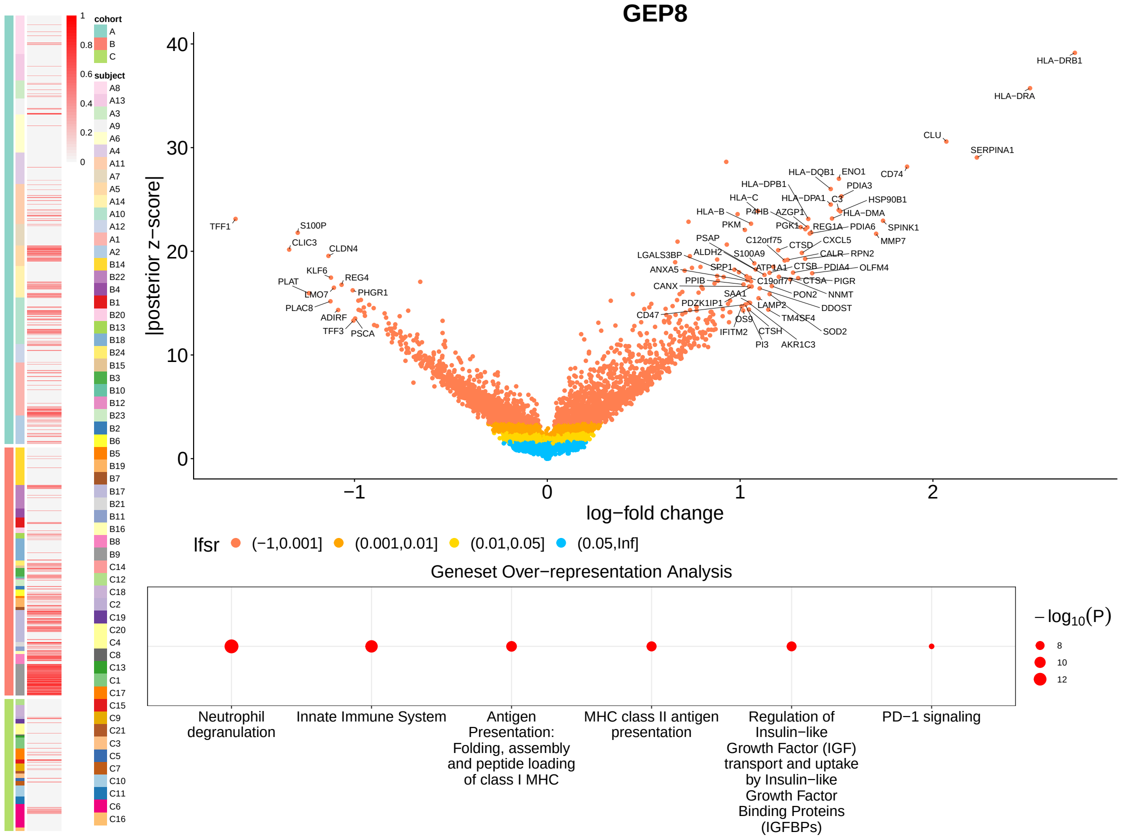
GEP8
cohort
A
B
C
subject
|posterior z−score|
log−fold change
lfsr
(−1,0.001]
(0.001,0.01]
(0.01,0.05]
(0.05,Inf]
HLA−DRB1
HLA−DRA
CLU
SERPINA1
HLA−DQB1
ENO1
CD74
HLA−DPB1
PDIA3
HLA−C
HLA−DPA1
C3
HSP90B1
HLA−B
P4HB
AZGP1
HLA−DMA
PKM
PGK1
REG1A
PDIA6
SPINK1
PSAP
C12orf75
CTSD
CXCL5
MMP7
LGALS3BP
ALDH2
S100A9
CALR
RPN2
ANXA5
SPP1
ATP1A1
CTSB
PDIA4
OLFM4
PPIB
C19orf77
CTSA
PIGR
CANX
SAA1
PON2
NNMT
PDZK1IP1
LAMP2
DDOST
CD47
OS9
TM4SF4
IFITM2
CTSH
SOD2
PI3
AKR1C3
TFF1
S100P
CLIC3
CLDN4
KLF6
PLAT
REG4
EMO7
PHGR1
PLAC8
ADIRF
TFF3
PSCA
Geneset Over−representation Analysis
Neutrophil degranulation
Innate Immune System
Antigen Presentation: Folding, assembly and peptide loading of class I MHC
MHC class II antigen presentation
Regulation of Insulin−like Growth Factor (IGF) transport and uptake by Insulin−like Growth Factor Binding Proteins (IGFBPs)
PD−1 signaling
−log10(P)

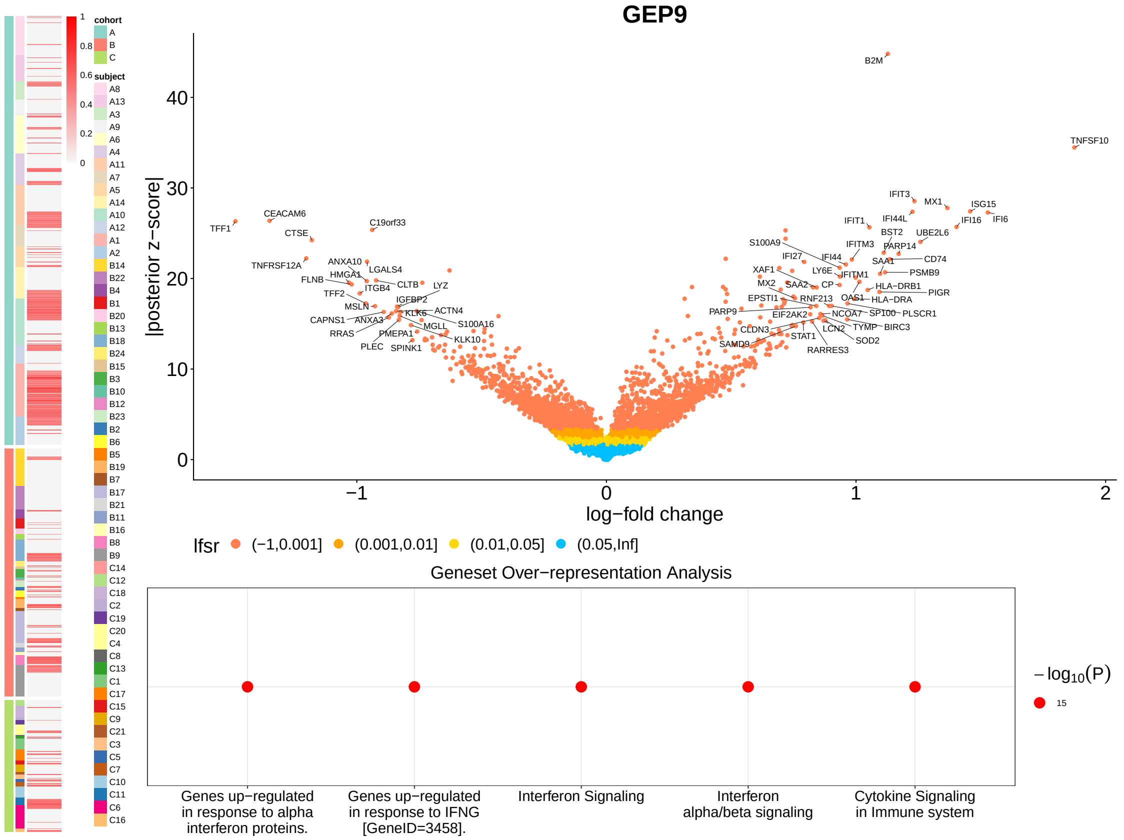

GEP9
|posterior z−score|
log−fold change
lfsr (−1,0.001] (0.001,0.01] (0.01,0.05] (0.05,Inf]
cohort
subject
Geneset Over−representation Analysis
Genes up−regulated in response to alpha interferon proteins.
Genes up−regulated in response to IFNG [GeneID=3458].
Interferon Signaling
Interferon alpha/beta signaling
Cytokine Signaling in Immune system
−log10(P)

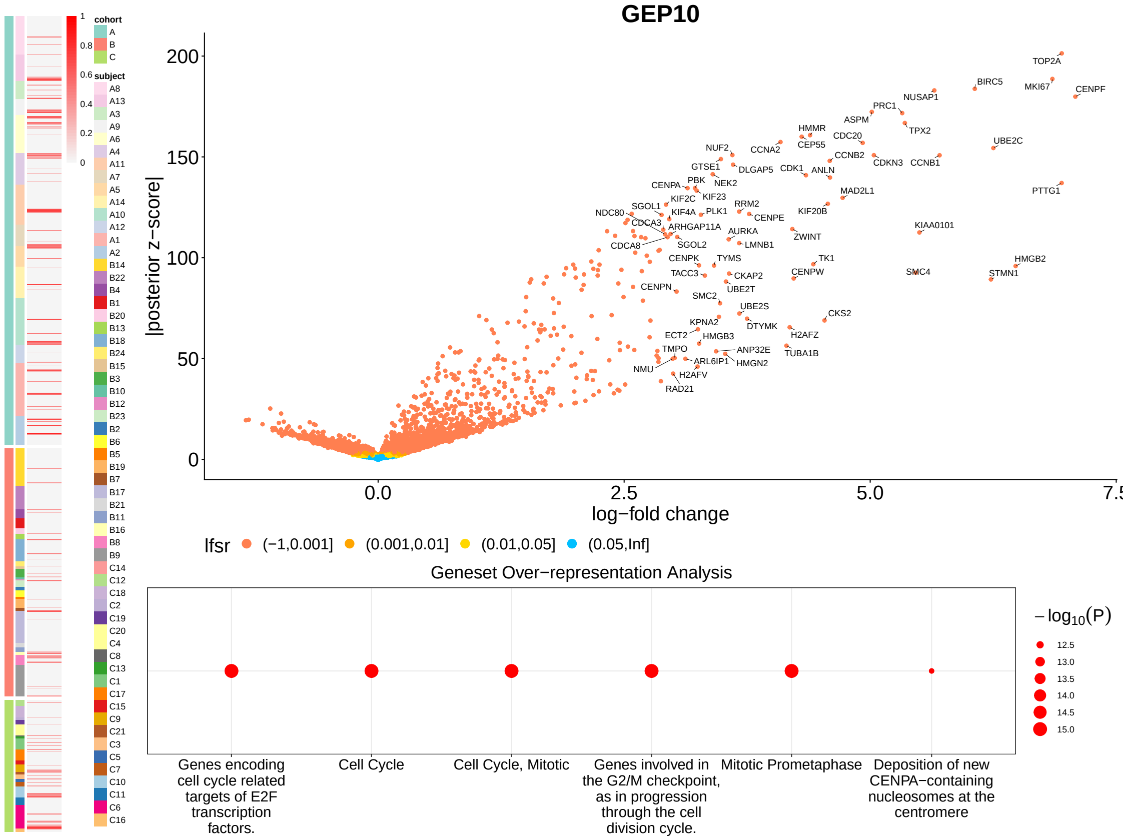

GEP10
|posterior z−score|
log−fold change
lfsr (−1,0.001] (0.001,0.01] (0.01,0.05] (0.05,Inf]
cohort
subject
Geneset Over−representation Analysis
Genes encoding cell cycle related targets of E2F transcription factors.
Cell Cycle
Cell Cycle, Mitotic
Genes involved in the G2/M checkpoint, as in progression through the cell division cycle.
Mitotic Prometaphase
Deposition of new CENPA−containing nucleosomes at the centromere
−log10(P)

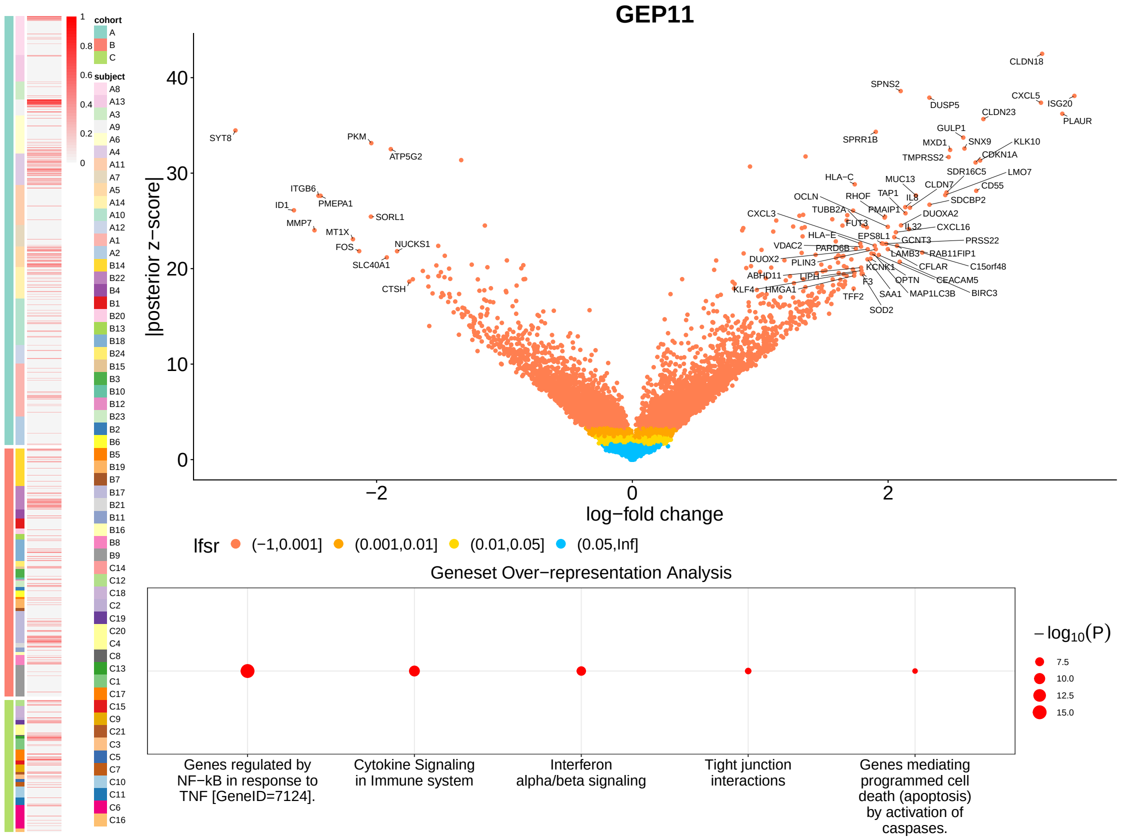
GEP11
cohort
A
B
C
subject
|posterior z−score|
log−fold change
lfsr (−1,0.001] (0.001,0.01] (0.01,0.05] (0.05,Inf]
Geneset Over−representation Analysis
−log10(P)
7.5
10.0
12.5
15.0
Genes regulated by NF−kB in response to TNF [GeneID=7124].
Cytokine Signaling in Immune system
Interferon alpha/beta signaling
Tight junction interactions
Genes mediating programmed cell death (apoptosis) by activation of caspases.

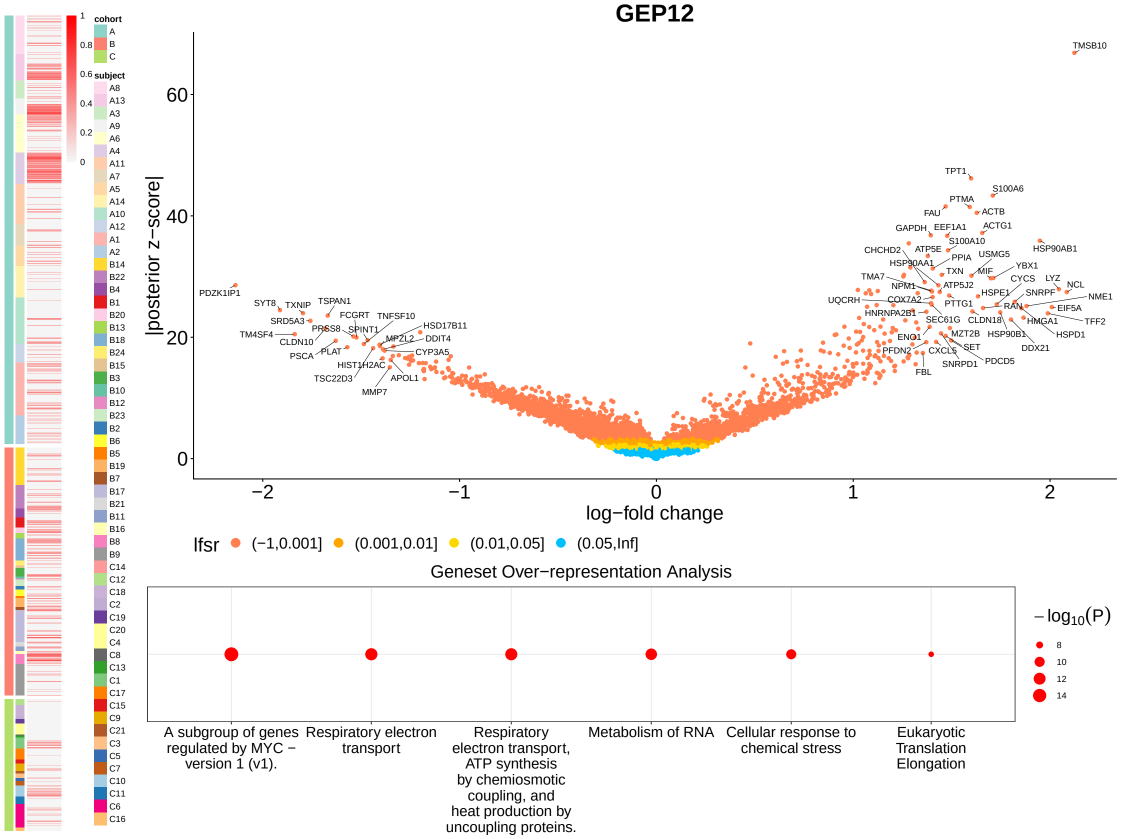
GEP12
cohort
A
B
C
subject
|posterior z−score|
log−fold change
lfsr (−1,0.001] (0.001,0.01] (0.01,0.05] (0.05,Inf]
Geneset Over−representation Analysis
−log10(P)
8
10
12
14
A subgroup of genes regulated by MYC − version 1 (v1).
Respiratory electron transport
Respiratory electron transport, ATP synthesis by chemiosmotic coupling, and heat production by uncoupling proteins.
Metabolism of RNA
Cellular response to chemical stress
Eukaryotic Translation Elongation

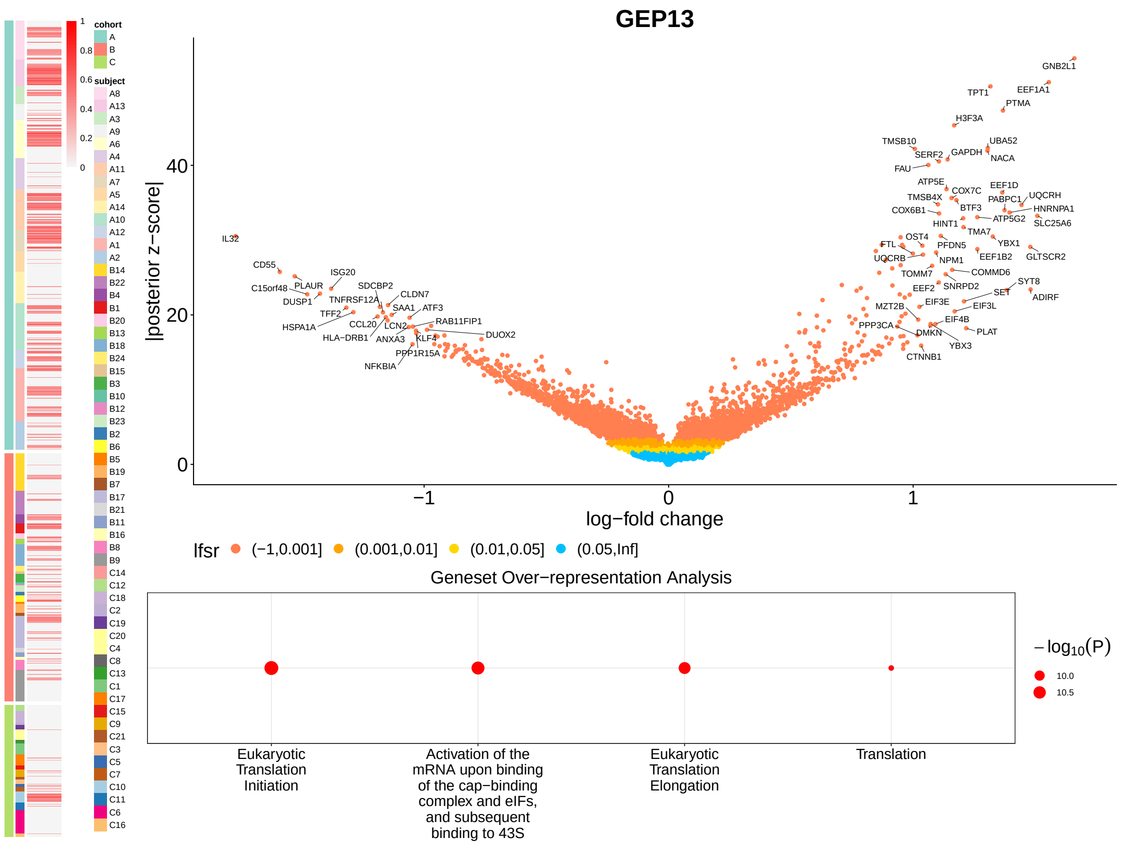

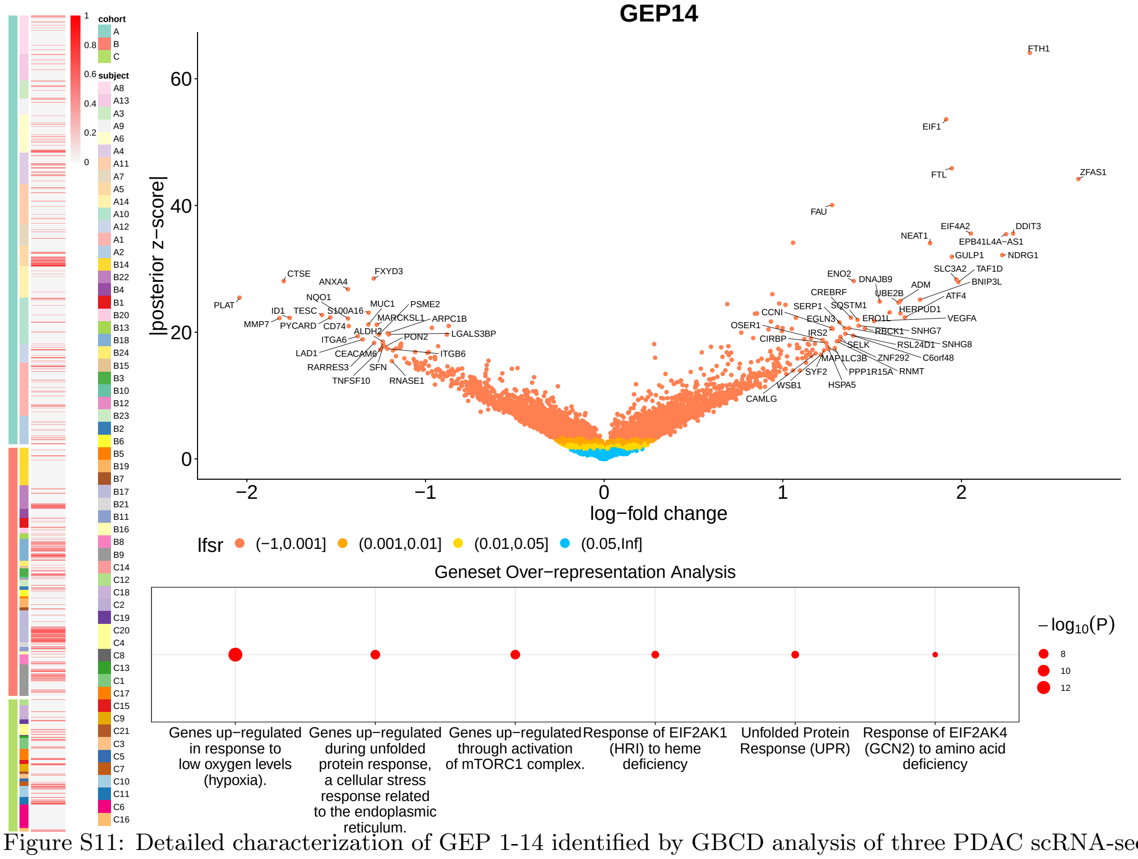

Figure S11: Detailed characterization of GEP 1-14 identified by GBCD analysis of three PDAC scRNA-seq datasets.
